# iDREM: Interactive visualization of dynamic regulatory networks

**DOI:** 10.1371/journal.pcbi.1006019

**Published:** 2018-03-14

**Authors:** Jun Ding, James S. Hagood, Namasivayam Ambalavanan, Naftali Kaminski, Ziv Bar-Joseph

**Affiliations:** 1 Computational Biology Department, School of Computer Science, Carnegie Mellon University, Pittsburgh, Pennsylvania, United States of America; 2 Division of Respiratory Medicine, Department of Pediatrics, University of California, San Diego and Rady Children’s Hospital, La Jolla, California, United States of America; 3 Division of Neonatology, Department of Pediatrics, University of Alabama at Birmingham, Birmingham, Alabama, United States of America; 4 Section of Pulmonary, Critical Care and Sleep Medicine, Yale School of Medicine, New Haven, Connecticut, United States of America; Hebrew University of Jerusalem, ISRAEL

## Abstract

The Dynamic Regulatory Events Miner (DREM) software reconstructs dynamic regulatory networks by integrating static protein-DNA interaction data with time series gene expression data. In recent years, several additional types of high-throughput time series data have been profiled when studying biological processes including time series miRNA expression, proteomics, epigenomics and single cell RNA-Seq. Combining all available time series and static datasets in a unified model remains an important challenge and goal. To address this challenge we have developed a new version of DREM termed interactive DREM (iDREM). iDREM provides support for all data types mentioned above and combines them with existing interaction data to reconstruct networks that can lead to novel hypotheses on the function and timing of regulators. Users can interactively visualize and query the resulting model. We showcase the functionality of the new tool by applying it to microglia developmental data from multiple labs.

This is a PLoS Computational Biology Software paper.

## Introduction

The analysis and modeling of dynamic regulatory networks remains a major goal of systems biology. Several methods for the analysis of such networks using a wide range of high throughput biological datasets have been developed over the last 15 years. Initial methods have mainly focused on using time series microarray data [1–3], though over the years these methods were extended by utilizing several other types of high throughput temporal and static data. Examples include methods that combine time series RNA-Seq and ChIP-Seq data [4, 5], methods for the analysis of epigenetic dynamics [6], microRNA regulation over time [7–9], time series proteomics [10–12] and, most recently, single cell RNA-Seq (scRNA-Seq) data [13, 14].

While each of the above data types has been studied and modeled on its own, relatively few methods have been developed to integrate multiple time series data types and we are not aware of any current method that can integrate all of them in a comprehensive analysis and visualization framework. In 2007, we presented the Dynamic Regulatory Events Miner (DREM) that was developed to integrate time series gene expression and static protein-DNA interaction data [15]. DREM learns an Input Output Hidden Markov Model (IOHMM) which attempts to identify bifurcation points—time points in which a set of genes that are co-expressed up to that point start to diverge. These points are then annotated by the transcription factors (TFs) that are predicted to regulate these genes allowing the method to assign dynamics to the (often static) protein-DNA interaction data. Over the years we have extended DREM so that it can utilize time series miRNA data [16], static ChIP-Seq data [17] and static protein-interaction data [18]. DREM has been widely used, by us and others, to model regulatory networks in a wide range of conditions and species [19–21].

**Fig 1:**
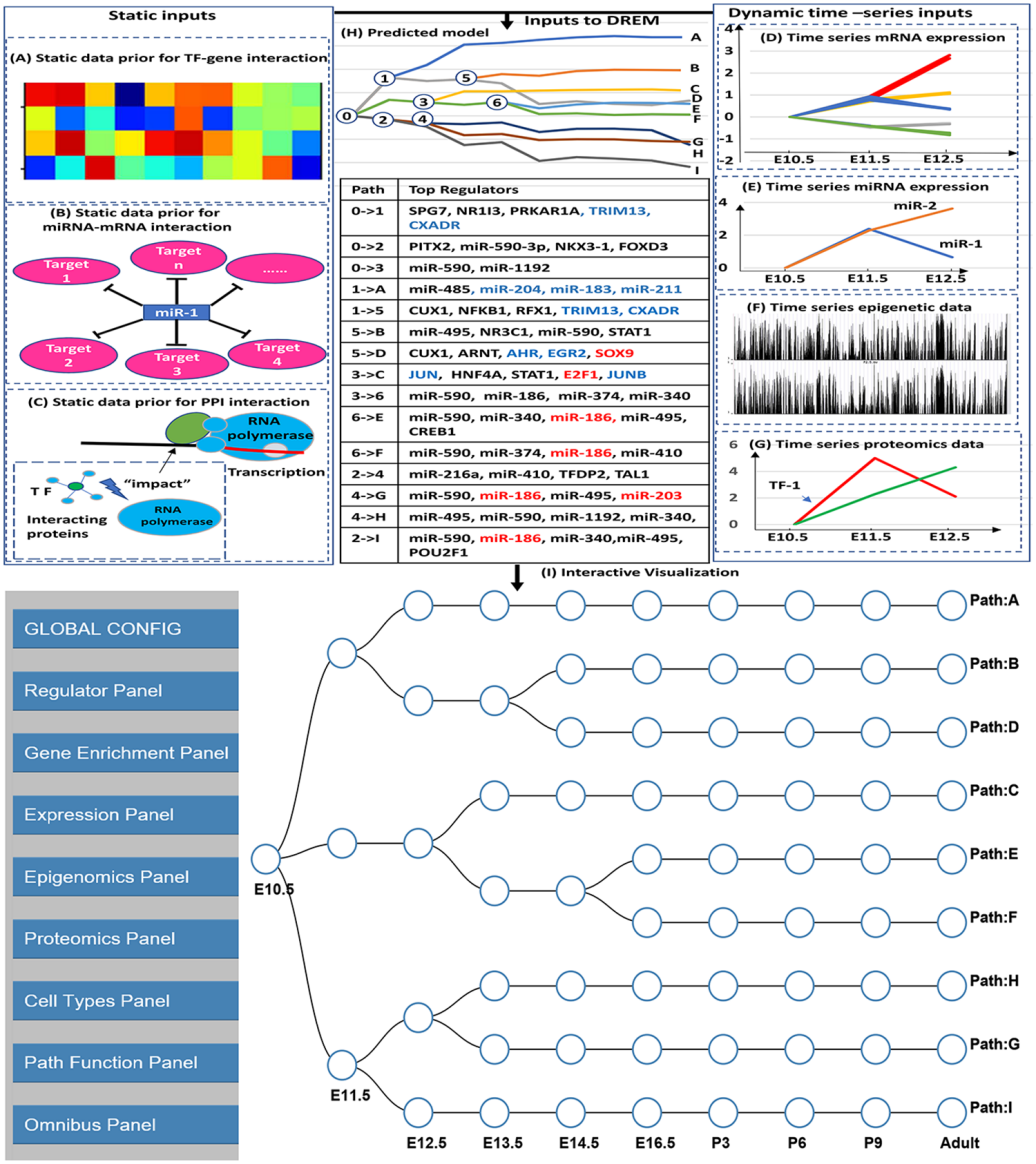
DREM and iDREM flowchart. **Top**: Data types integrated to learn the DREM model include general, static interaction data (**A**) Transcription factor (TF)-gene interaction; (**B**) miRNA-mRNA interaction; (**C**) protein-protein interaction (PPI) and condition specific time series data (right): (**D**) mRNA expression; (**E**) miRNA expression; (**F**) Epigenetic data; (**G**) Proteomics data. The resulting model (**H**) provides a summary of different gene groups in the experiment, their expression level, their temporal profiles and the regulators (TFs and miRNAs) that control different bifurcation events the. **Bottom I**: The iDREM representation of the learned DREM model above. Note that this representation removes the actual levels and only provide a schematic view for the paths and splits in the model. The actual expression levels and several other aspects of the model and the data can be interactively viewed by using the various panels available (left).

While useful, DREM and its extensions are still unable to utilize several recent high throughput time series data types. These include epigenetic data (methylation, histone modification etc.), time series proteomics datasets and time series scRNA-Seq data. While past studies have usually profiled only one of these data types, more recent work often profiles multiple data types over time [22] which necessitates methods that can combine all of these in a single analysis and visualization framework. In addition, the current DREM output is a dynamic network figure (Fig 1) which does not allow for interactive analysis of the resulting model. To address these issues we developed the interactive DREM (iDREM) tool that provides support for more data types and greatly improves the visualization allowing users to interactively query the reconstructed network. We also allow users to project scRNA-Seq data on the resulting model helping highlight the relationships between different cell types and the trajectories observed in bulk expression analysis (S3 Fig).

## Design and implementation

In previous DREM versions ([15, 17, 18]), we discussed the integration of time-series mRNA expression, time-series miRNA expression and static TF-gene and protein-protein interaction data. Here we focus on the new capabilities of iDREM including the ability to utilize time-series proteomics, epigenomics, and scRNA-Seq data and the interactive visualization options.

### Incorporating time series proteomics data

We use the proteomics data to improve our ability to detect the *time* of TF activation. In previous versions of DREM we used a static, prior regulatory interaction matrix (inferred from previous experiments not necessarily related to the condition being studied). To obtain a dynamic version of such matrix we do the following. First, if a TF protein is highly expressed at a specific time point we increase the prior on its activity for that time point. Second, to account for post-translational modifications which are not always reflected by the protein levels we also use protein interaction information. Specifically, for each TF we look at the average expression of its known interaction partners at each time point. If the levels of proteins that interact with the TF are increased (decreased) we increase (decrease) the prior on that TF for that time point by adjusting the values in the prior regulation matrix for that TF. See S1 Text for complete details. The interactive visualization (S1 and S2 Figs) further supports exploration of the proteomics data and its impact. Users can view the protein levels of the specific genes and TFs. To determine the impact of the proteomics data, users can run iDREM with and without this data and directly compare the resulting models.

### Utilizing time series epigenomics data

**Fig 2:**
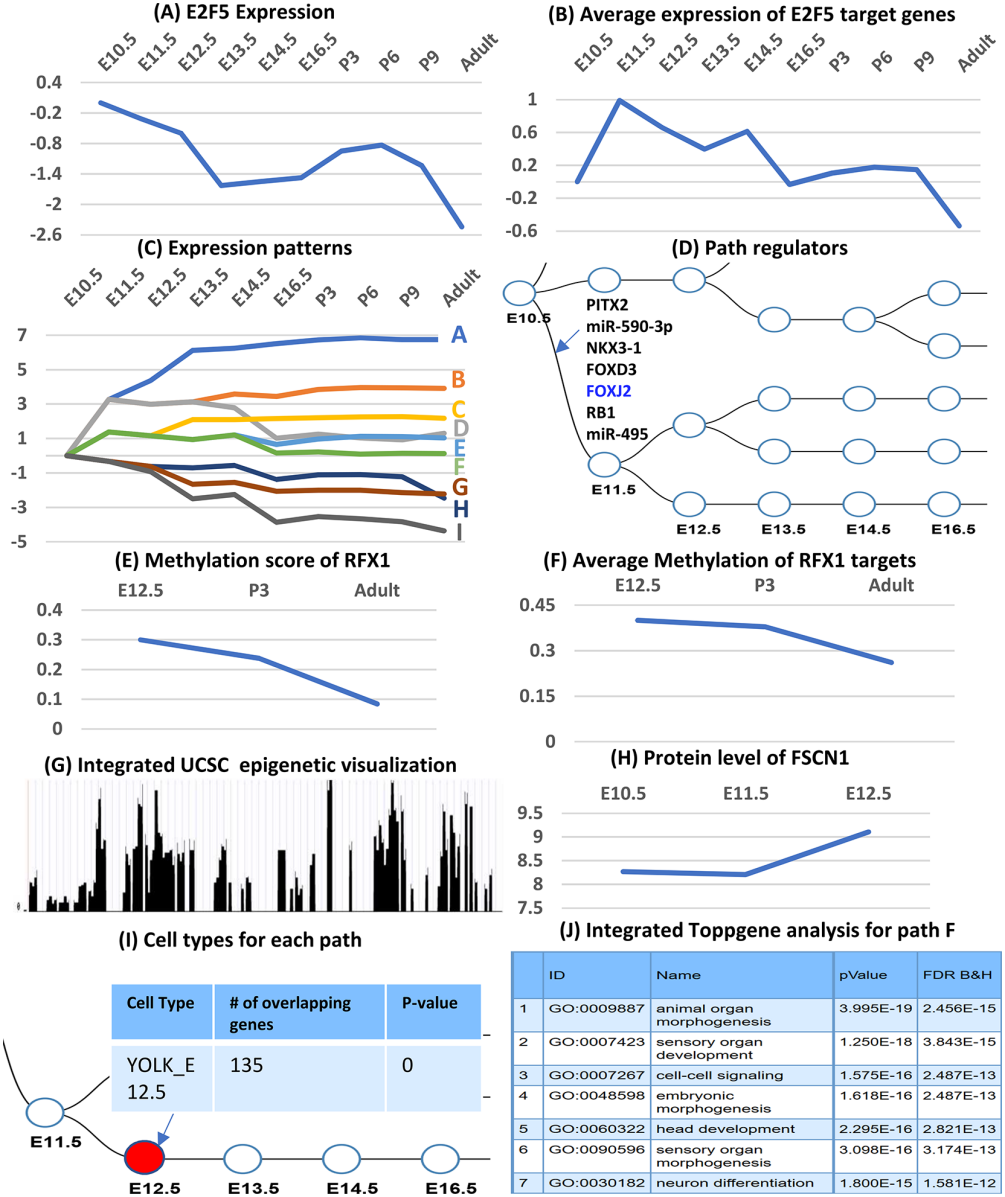
iDREM visualization functions. **Top**: Expression of a regulator (E2F5) (**A**) and its targets (**B**). **2nd row**: Expression patterns (similar to the original DREM result, can be viewed from the tool as well) (**C**) and the regulators for each of these splits (**D**). **3rd row**: Methylation of a regulator (**E**) and its targets (**F**). **4th row**: Integration with additional browsers for viewing epigenetic data for specific TFs / genes (**G**) and protein level for specific TFs/proteins (**H**). **5th row**: Intersection of path genes with single cell data (**I**) and integrated GO functional analysis (**J**).

iDREM adds support for dynamic epigenetic data. Here we discuss time series histone methylation (H3K4me2) data, though iDREM supports other types of epigenetic data as well (S1 Text). Epigenetic data is used to further improve our ability to assign temporal activity to TFs. Specifically, depending on the type of time series data that the user provides, iDREM either increases or decreases the prior on the likelihood of binding of a specific TF to each of its targets. For example, H3K4me2 methylation is associated with “activation” [23], and thus we use it to increase the likelihood of binding in cases where a TF binding site is methylated for a specific target at a specific time point. See S1 Text for details on how the epigenetic data is used and integrated into the IOHMM learning process. Additionally, iDREM provides a number of options for visualizing epigenetic data and its relationship with other data types. For genes, users can plot the temporal profiles of their promoters and explore the overall impact of the epigenetic data on targets of specific TFs/ miRNAs. Users can also explore the difference in epigenetic scores between two time points and can view the data directly on the UCSC genome browser [24] (Fig 2(G)).

### scRNA-Seq and sorted cell data

A new and exciting type of high-throughput time series data is available from experiments that profile the expression in single cells (e.g. scRNA-Seq) [13]. Other studies have profiled different types of homogeneous cells over time [25, 26] (often termed sorted cells). To enable the integration of single and sorted cell data with bulk studies iDREM allows users to superimpose cell type studies on the reconstructed models. This is performed using the “Cell Types” panel which allows users to upload single cell data (for specific time points) and then intersects the top differentially expressed (DE) genes in these datasets with genes assigned to nodes that represent the same time points in the iDREM model. This enables users to determine the cell type composition of the different nodes and paths and to infer whether specific changes observed are related to activation of TFs in existing cells or the formation of new cell types.

### Interactive visualization of the reconstructed model

In addition to visualizing the new data types discussed above, several additional panels are provided for users to explore the reconstructed model, trajectories and interactions of specific TFs, genes and miRNAs. The panels are shown in S1 Fig. They include the “Global Config panel” which provides general functions for the appearance of the schematic network. The “Expression panel” allows users to interactively look at the expression of specific genes, sets of genes and miRNAs (Fig 2(A)) and determine the path they were assigned to. The “Regulator panel” allows users to determine regulators for specific splits (Fig 1) and paths. It can also be used to determine all paths controlled by a specific TF or miRNA. Users can change the setting to only select those paths for which the regulator is one of the top *X* regulators (where *X* is user defined) or based on the assigned p-value. See S1 Text for complete details on all panels.

### Implementation

iDREM is implemented using a combination of Java and Javascript. The regulatory model prediction part is implemented in Java and the interactive visualization is implemented in Javascript with D3.js and Google charts. Users only need to run the main java program idrem.jar to get all results including the interactive visualization. Please refer to iDREM readme for more details (https://github.com/phoenixding/idrem).

## Results

### Applying iDREM to study mouse microglia development

We illustrate the functionality of iDREM by applying it to reconstruct mouse microglia developmental regulatory networks from a diverse set of high throughput biological data types (S1 Table). Microglia are a type of small macrophage-like glial cell and these cells comprise up to 15% of all cells in the brain. Most of the data we used for this analysis, including mRNA expression data, histone methylation data and single cell RNA-Seq data is from a study of microglia development [22]. We have also included whole brain time series proteomics data [27] and miRNA expression data [28]. While the whole brain data may only partially overlap with the microglia profiles, since the focus here is on the methods and visualization, we have added that data to fully showcase the ability of iDREM to integrate and interactively visualize diverse types of time series data.

The datasets overlapped in some of the time points used (S1 Table) though the overlap was only partial. This highlights another advantage of iDREM, the ability to utilize some data types in only a subset of time points which can improve the ability of researchers to integrate their data with other, publicly available, data. In addition to the condition-specific, time series data sets iDREM also uses general static TF-DNA interactions data similar to DREM 2.0 [17], static miRNA-mRNA interactions data [16] and protein-protein interactions data which are used for the time series proteomic data analysis and were downloaded from STRING(V10.5) [29].

Fig 1 provides an overview of the data used by iDREM to reconstruct the networks, the resulting DREM model and a screenshot from the interactive visualization tool (S2 Fig). The model determines the different paths and splits, the genes assigned to them and the TFs and miRNAs that control each of the paths and splits. The model reconstructed for the microglia development data (Fig 1) includes 9 different paths, which have each been assigned a set of regulating TFs and miRNAs. Several of the paths are correctly enriched for GO functions related to immune defense and development of the central nervous system, which have been reported as the primary function of microglia cells [30]. S2 Table presents the top GO terms associated with each path.

Several of the regulators identified for the paths are known to regulate microglia development (S3 Table). Specifically, the reconstructed network includes 5 of the 7 TFs identified manually in the original microglia study [22], all of which are determined to be very significant. In addition, the method identified a number of additional microglia relevant TFs including CD40 which is known to be a microglia marker [31], SMAD1 which is an immune system factor [32], TRAF4 which is reported to be involved in multiple immune functions [33] and more. Fig 1H presents many of the top TFs and miRNAs identified by iDREM as controlling the various paths in the model.

Fig 2 displays some of the visualization capabilities of iDREM. It also shows how the new functionality improves the accuracy of the reconstructed model. For example, regulatory factor X1 (RFX1) is an immune response factor [34], consistent with the function of microglia cell. However, without the time series methylation data RFX1 cannot be identified as a regulator. The large increase in the activation prior for RFX1 (Fig 2(E)) leads to much higher probability that RFX1 is regulating path B resulting in its inclusion in the reconstructed model. Note, The TF binding prior is smaller for genes with larger methylation score in iDREM model (might need a pre-processing for methylation associated with increased TF binding activites such as H3K4me2 methylation, please refer the iDREM manual for details). Similarly, the elevated protein expression levels of fascin actin-bundling protein 1 (FSCN1), an immune system regulator [35], enabled iDREM to correctly identify it as controlling the path from E12.5 to E13.5 (Fig 2(H)).

In this study, we provided some anecdotal evidence for the impact of these newly introduced features such as proteomics and epigenetics data (in Fig 2). We also performed additional analysis in which we removed one data type at a time and analyzed the differences in the resulting networks, significant GO functions associated with different paths and the set of regulators identified by the models. Specifically, we compared the 4 iDREM models: I) Does not use any of the new datasets (only uses miRNA, mRNA expression and the static interaction data); II) the data used by I + the time series proteomics data; III) the data used by I + the time series methylation data; IV) The model presented in the paper that uses all data types. We see an improvement when using more data types and the best results are obtained by model IV indicating that including all data types can lead to more accurate models. Please refer to S1 Text, S4, S5, S6, S7 and S8 Figs and S4 Table for the complete details.

## Availability and future directions

The iDREM code and software, with an example input dataset and detailed instructions are available from GitHub (https://github.com/phoenixding/idrem). All the data, code and results are also available at the supporting website (http://www.cs.cmu.edu/~jund/idrem/). Future work of iDREM will focus on better integration of new data (e.g. time series Single-cell ATAC-Seq).

## Supporting information

S1 Text Supporting methods and results. This file provides the detailed method description and also the supporting results.
(PDF)

S1 Fig iDREM visualization configuration panels. (A) Global config, which can be used to customize the visualizations (e.g. background color, node color, visualization size). (B)Regulator Panel, which can be used to visualize the gene/miRNA expression. (C) Enrichment panel, which an be used to find the enriched paths/nodes in iDREM model for any given inputs. (D)Expression panel, which can be used to visualize gene or miRNA expression. (E) Epigenomics Panel, which can be used to explore and visualize the epigenomics data used in the study. (F) Proteomics Panel, which can be used to visualize the protein levels. (G) Cell Types Panel, which can be used to explore/visualize the Single-cell or Sorted-Cell data. (H) Path Function Panel, which can be used to visualize the associated GO functions and regulators for each path. (I) Omnibus Panel, which can be used to explore and visualize the TF/gene in all possible panels. For a more detailed description, please refer to iDREM manual.
(PDF)

S2 Fig iDREM interactive visualization. This figure shows the interactive visualization for the microglia data used in the study.
(PDF)

S3 Fig An example of using single-cell RNA-seq data in iDREM. (A) The single-cell RNA-seq data. (B) Cluster the cells into different sub-types based on the expression profile. (C) Identify the signature genes (marker genes) for each cell type. (D) Intersect the marker genes (of specific cell-type) with the predicted paths/nodes in iDREM model to identify enriched paths/nodes. (E) This enables users to determine the cell type composition of the different nodes and paths and to infer whether specific changes observed are related to activation of TFs in existing cells or the formation of new cell types.
(PDF)

S4 Fig Predicted paths for models I, II, III, IV. I: only use miRNA and mRNA expression data; II: data used by I + time series proteomics data; III: the data used by I + the time series methylation data; IV: using all data presented in the study.
(PDF)

S5 Fig Sankey Diagram for model I. The Sankey Diagram shows the GO functions and regulators associated with each of the predicted paths.
(PDF)

S6 Fig Sankey Diagram for model II. The Sankey Diagram shows the GO functions and regulators associated with each of the predicted paths.
(PDF)

S7 Fig Sankey Diagram for model III. The Sankey Diagram shows the GO functions and regulators associated with each of the predicted paths.
(PDF)

S8 Fig Sankey Diagram for model IV. The Sankey Diagram shows the GO functions and regulators associated with each of the predicted paths.
(PDF)

S1 Table Mouse microglia development time points used in this paper. (PDF)

S2 Table Top Go Terms associated with each path. (PDF)

S3 Table Supported regulating factors predicted by iDREM. (PDF)

S4 Table Regulator comparison for models using different sets of input data. (PDF)
