# Supplementary material for: iDREM: Interactive visualization of dynamic regulatory networks
Source: PLoS Comput Biol. 2018 Mar 14;14(3):e1006019. doi: 10.1371/journal.pcbi.1006019 (PMC5868853; doi:10.1371/journal.pcbi.1006019)
Supplement: S1 Text — This file provides the detailed method description and also the supporting results. (PDF) [file pcbi.1006019.s001.pdf]

# Supplements for iDREM: Interactive visualization of dynamic regulatory networks

Jun Ding, James Hagood, Namasivayam Ambalavanan, Naftali Kaminski, and Ziv-BarJoseph

## Contents

**Supporting Results** **2**
Interactive visualization of the reconstructed model . . . . . . . . . . . . . . . . . . . 2
Global config panel . . . . . . . . . . . . . . . . . . . . . . . . . . . . . . . 2
Expression panel . . . . . . . . . . . . . . . . . . . . . . . . . . . . . . . 2
Regulator panel . . . . . . . . . . . . . . . . . . . . . . . . . . . . . . . 3
Epigenomics panel . . . . . . . . . . . . . . . . . . . . . . . . . . . . . . 3
Proteomics panel . . . . . . . . . . . . . . . . . . . . . . . . . . . . . . 4
Cell Types panel . . . . . . . . . . . . . . . . . . . . . . . . . . . . . . 5
Functional and user provided list enrichment panel . . . . . . . . . . . . . . . . 6
Omnibus panel . . . . . . . . . . . . . . . . . . . . . . . . . . . . . . . 6
Assessment of the contribution of each data type to the predicted model . . . . . 6

**Supporting Methods** **8**
Underlying probabilistic framework presented in DREM . . . . . . . . . . . . . . 8
Integrating proteomics and protein-protein interactions . . . . . . . . . . . . . . . 8
Integrating epigenomic data . . . . . . . . . . . . . . . . . . . . . . . . . . 10
Inferring dynamic input: TF-DNA regulation map . . . . . . . . . . . . . . . . . . 11
Likelihood density function . . . . . . . . . . . . . . . . . . . . . . . . . . 12
Model learning . . . . . . . . . . . . . . . . . . . . . . . . . . . . . . . . 14

**Supporting Tables** **15**

**Supporting Figures** **17**

# Supporting Results

## Interactive visualization of the reconstructed model

We next discuss the additional features provided by the interactive visualization tool, which includes support for new data types (epigenomics, proteomics and single cells) and several panels that enable users to explore the different data types used for constructing the model, how each affects the learned model and how they relate to each other. The visualization control configuration panels are shown in Supporting Figure 1.

### Global config panel

Supporting Figure 1 (A) shows the global configuration panel which provides general functions for the appearance of the schematic network. Users can always press the "Reset" button to reset all configuration changes. The slider allows users to zoom in and out enlarging the areas they are interested in. Users can also customize the visualization background color, node color, text color and path colors. In addition to overall appearance users can also customize the ways clicks are interpreted. It can be customized so that a click presents properties which are available in the dropdown menu: Regulator, Average methylation for all top regulator targets, Average methylation for genes in node etc. For example, by selecting the "Regulator" option, users can see the regulators (both TFs and miRNAs) for the edge ending at the node they clicked. Similarly, the users can also customize the ways that shift+clicks are interpreted. (the manual provides an explanation for all other possible functionality of clicks).

### Expression panel

The reconstructed models are presented using a schematic tree structure as shown in Supporting Figure 2. The expression panel allows users to interactively look at the expression of specific genes, set of genes and miRNAs. In the panel (Supporting Figure 1 (D)), a user can search for the path and expression of a specific gene/miRNA using the "Explore Gene", "Explore miRNA", "Explore Gene/miRNA absolute expression" and "Explore Regulator target expression". Users can query the gene expression using "Explore gene", query the miRNA expression using "Explore miRNA". In addition, users can click on the "Explore Regulator target expression", to see the profiles of a set of genes predicted to be regulated by a specific TF or miRNA and the paths they were assigned to. Users can choose the expression visualization in the form of a Line chart, a Bar chart or Column chart. We also implemented a "show Path expression" function, which can be used to visualize the predicted model using the actual expression, in which the geometric position of the path represents the actual path expression (e.g. higher the path position, larger the expression).

**Regulator panel**

A key advantage of DREM and its extensions is the ability to assign dynamic information to static interaction data. To determine regulators for specific splits (Supporting Figure 2) and paths, users can hover the mouse on a node or click a specific node to obtain a table of its top regulators. However, researchers may also be interested in all paths controlled by a specific TFs or miRNA. For this, the regulator panel (Supporting Figure 1 (B)) allows them to select a regulator and see all paths / nodes it regulates. Users can change the setting to only select those paths for which the regulator is one of the top $X$ regulators (where $X$ is user defined) or based on the regulation p-value. The regulated paths/nodes for the selected regulator are highlighted in blue. The tool also provides a list of all regulators in the dropdown menu for users to select from.

**Epigenomics panel**

We have added support for histone methylation(H3K4me2) data as an example of epigenomic data, which is used to further improve our ability to assign temporal activity to TFs. Specifically, depending on the type of time series epigenomic data that the user provides, we can either increase or decrease the prior on the likelihood of binding of a specific TF to its target. For example, DNA methylation is associated with repression, which inhibits the TF binding. H3K4me2 methylation is associated with "activation", which implies potential TF binding. Thus, unlike prior versions of DREM, in iDREM we use time point specific interaction information when computing regulators for specific splits. See Methods for details on how the epigenomics data is used and integrated into the IOHMM learning process. To illustrate the usefulness of the epigenomic data for reconstructing accurate models consider the regulatory factor X1 (RFX1) TF. RFX1 has been known to be involved in immune response [1], consistent with the function of microglia cells [2]. However, without the methylation data RFX1 is not identified as a regulator for this path. RFX1 H3K4me2 methylation significantly increased after E12.5 (note that the methylation score represents the repression score, which is decreasing for RFX1, see methods). The large increase in the activation prior for RFX1 leads to much higher probability that RFX1 is regulating path B resulting in its inclusion in the reconstructed model.

In addition to using the epigenomic data to improve the model learning, we also provide a number of options for visualizing the data itself and its interactions with the other data types used in the model. This type of analysis is available from the methylation panel (Supporting Figure 1 (E)). For genes, we plot the temporal methylation profiles of their promoters using the "Explore gene" button. Please note that the "methylation" here can be used to represent any type of epigenomic data. If the epigenomic data is associated with "repression" (e.g. DNA methylation), the methylation plot represents the score itself. If the

epigenomic data is associated with "activation" (e.g. H3K4me2 histone methylation), the methylation plot represent the opposite of the score (Larger scores in the methylation plot represent smaller original methylation score). In other words, different types of epigenomic data will be treated differently in the DREM model, please refer to the method section for complete details.

We also provide ways to explore the overall methylation of targets of specific TFs/ miRNA using the "Explore Regulator Methylation Targets in node". Users are also able to narrow down the regulator targets that are combined, for example by focusing only on targets that are assigned to a specific node. Users can also explore the difference in epigenomic scores between two time points (for example, the different levels of specific gene or a set of genes) using the button "Explore methylation difference" and a dropdown menu to select the time points. epigenomic scores can be visualized using Column chart, Bar chart or Line chart. To enable exploring the epigenomic scores using genomic locations, we also enable users to view the data directly on the UCSC genome browser [3].

### Proteomics panel

In the current version, we also provide support for the integration of time series proteomics data. In prior DREM models [4], the activity of the TF at the different time points was determined by the activity of its predicted targets. As mentioned above, in this version we also use epigenomic data for such inference and, when available, we also rely on proteomics data. This data is used in two ways. First, if we identify a TF whose protein is highly expressed at a specific time point we increase the prior on its activity for that time point (encoded by the prior regulatory interaction matrix mentioned above). Second, to account for post-translational modifications which are not always reflected by the protein levels (i.e. a protein can be activated even if its expression remains the same) we also use protein interaction information. Specifically, for each TF we look at the average expression of its known interaction partners at each time point. If the levels of proteins that interact with the TF is increased (decreased) we increase (decrease) the prior on that TF for that time point by adjusting the values in the prior regulation matrix for that TF. See Methods for complete details on how the changes in priors are determined based on the time series proteomics data. Use of the proteomics data can improve the ability of the DREM method to correctly identify the set of active TFs at different time points. For example, as can be seen in main text Figure 2H, the protein expression of fascin actin-bundling protein 1 (FSCN1), an immune system regulator [5], significantly increases at E13.5 which enables iDREM to identify it as a regulator for the path from E12.5 to E13.5.

In addition to using the proteomics data for constructing the model, we have also provided support to explore the data and its impact in the interactive visualization tool. In the

proteomics panel (Supporting Figure 1 (F)), users can view the protein levels of specific genes and TFs using the "Explore protein level" button. Similar to the expression panel, users can choose bar chart, column chart or line chart for visualization. We also provide support for running iDREM with and without the proteomics data and comparing the results to determine the impact of this data on the resulting model.

### Cell Types Panel

A new and exciting type of high-throughput time series data is available from experiments that profile the expression in single cells (e.g. scRNA-Seq) [6]. Other studies have profiled different types of homogeneous cells over time [7, 8] (often termed sorted cells). Still, most available time series data is from bulk tissue experiments. To enable the integration of single and sorted cell data with bulk studies we have added to iDREM functions that allow users to superimpose cell type studies on the DREM models. While we usually assume that the bulk data contains the single cell/sorted cell data as part of the sample, iDREM does not require that this would be the case. What we do with the Single Cell or sorted cell data is to project it on the resuling model by computing the significance of the overlap between Single Cell / sorted cell differentially expressed genes and genes assigned to the nodes in the model at the relevant time point. Using this, researchers can observed whether Single Cell / sorted cell data is confined to a specific path /node or whether it spreads over multiple nodes and use that to assign TFs and functions to the Single cell data. They can also compare different cell types based on the nodes/paths they are assigned to and draw conclusions regarding common or unique pathways associated with the different cell types and, potentially, cross talk between them. Please refer to supporting figure 3 for an example single-cell analysis. This example shows the projection of Single cell neuronal development data on the microglia model, As can be seen, the set of marker genes identified for neuron cells based on the Single-cell experiments match path F of he microglia model (pink in the supporting figure). This is in good agreement with the GO analysis for that path that identified "nervous system development" as a significant function associated with path F.

This above anlaysis is visualized using the "Cell Types" panel as shown in Supporting Figure 1 (G). This panel allows users to upload single cell data (for specific time points and cell types) and then it intersects the top differentially expressed (DE) genes in these datasets with genes assigned to nodes that represent the same time points in the iDREM model. This enables users to determine the cell type composition of the different nodes and paths and to infer whether specific changes observed are related to activation of TFs in existing cells or the formation of new cell types. As an example, we have intersected the model in Supporting Figure 2 with yolk single cell data from E12.5. Results are shown in main Text Figure 2(I) in which a specific path (path I) is significantly overlapping with genes that are DE in yolk cells.

Path I is the most down regulated path in the model and this agrees well with observation in the original paper [9] that also noted that signature yolk genes, including Pf4 and Dab2 are down regulated at that time point.

#### Functional and user provided list enrichment panel

Users are able to view the functional enrichment for each node or path by shift clicking the node using the global config panel settings. Two different gene functional analysis methods Toppgene [10] and PANTHER [11] are provided for users to choose from the shift-click dropdown menu. In addition, in the Path function panel (Supporting Figure 1 (H)), we have implemented a Sankey diagram view for the iDREM model. This diagram provides joint visualization of the regulating factors (miRNA and TFs) and GO terms associated with each of the paths and the relationships between paths based on these terms. Specifically, in these diagrams the middle nodes represent the paths, the right nodes are the corresponding GO terms and the left nodes are the regulators. Users can view the Sankey diagram using the "show path function Sankey diagram" button and a slider is provided to adjust the GO term cutoff for the display. From the Sankey Diagram, we observed that many top paths (A,B,D,F) are associated with cell communication, nervous system development functions, which are supported by previous studies [12].

In addition to established gene annotation databases, users often have other lists of genes they may be interested in (specific diseases, specific mutation etc.). The gene enrichment panel (Supporting Figure 1 (C)) allows users to interactively search for paths and nodes that are enriched for genes in such lists. Users can upload a list of genes using any of the following separators: comma, tab, space and newline and the results are displayed on the iDREM model using different shading for nodes based on their enrichment.

#### Omnibus panel

In addition to data type based panel, the omnibus panel (Supporting Figure 1 (I)) provides a gene or TF centric view of all types of data. By inputting a specific TF name, the tool displays all activity related to this TF including its gene and protein expression levels, expression and epigenomic profiles of its targets, paths it regulates etc. This is performed by selecting the TF and clicking the "ShowOmnibus" button.

## Assessment of the contribution of each data type to the predicted model

Since the ground truth is often now known for most dynamic regulatory networks, including the microglia developmental networks discussed in this paper, it is challenging to perform

systematic analysis for the accuracy provided by the addition of each of the features (data type) used by iDREM. In the main manuscript, we provided some anecdotal evidence for the impact of these features. Besides, we also performed additional analysis in which we removed on data type at a time and analyzed the differences in the resulting networks, significant GO functions associated with different paths and the set of regulators identified by the models. Specifically, we compared the 4 iDREM models: I)Does not use any of the new datasets (only uses miRNA,mRNA exprssion and the static interaction data; II) the data used by I + the time series proteomics data; III) the data used by I+ time series epigenomics data (histone methylation in this study); IV) The model presented in the paper that uses all data types.

For each of these models, we looked at 1) the predicted paths; 2) significant GO functions associated with each path and; 3) regulators assigned to these paths. The models are presented in Supporting Figure 4.

As can be seen, while the predicted paths in the resulting models are similar (since they largely rely on the expression levels used by all models). When using additional information (models II-IV), more regulatory (splits) events are identified. Specifically, iDREM identifies 6 paths for model I, 8 paths for model II, 7 paths for model III and 9 paths for model IV. Thus more data types can lead to better separation of (similar) expression trajectories.

Sankey diagram for all 4 models with GO and regulator information are provided as Supporting Figures 5, 6, 7 ,8.

Again, while the set of significant functional annotations overlaps between the different models, we observe differences when increasing the types of data used by iDREM. Specially, for model I only two paths (A,D) are associated with function that are known to be related to microglia development (mainly immune response and development). In contrast, model II and III have 3 paths significantly enriched for these functions while model IV has 4 (A, B, E, F). In addition, comparing GO terms that are significantly associated with models IV and I, indicates that model I is enriched for relatively general functions while model IV is enriched for more specific developmental and immune response. For example, "positive regulation of defense response" and "cytokine-mediated signaling pathway" (very specific immune related functions) are identified in model IV but not in model I, in which only the general nervous system development function is identified as the immune related function.

As for the regulators, based on the microglia study from which we obtained the expression data (PMID27338705 and other related studies (see main text Results), we identified 10 microglia factors: Rxrb, Fli1,Egr1, Fos, Mafb, Mef2a, Jun, Cd40, Smad1, Traf4. The supporting table 4 lists these and whether they were assigned to paths in each of the model tested. Again, we see an improvement when using more data types and the best results are obtained by model IV indicating that including all data types can lead to more accurate models.

# Supporting Methods

We introduce a new method to learn a dynamic regulation map of TF and miRNA over time that jointly considers protein-DNA, miRNA-gene, time series gene expression, time series miRNA expression, time series epigenomic data and time series proteomics. The details of the method are presented in the remaining paragraphs of this section.

## Underlying probabilistic framework presented in DREM

DREM [4, 13, 14] integrates static, general TF-gene binding interactions, time-series gene expression and time-series miRNA expression data to determine the set of TFs and miRNAs that control gene expression over time. DREM is able to use the expression level of TFs to influence the learning of the classifier in the input-output hidden Markov model (IOHMM). The rationale behinds this is that TFs that are over- or under-expressed between time points have a higher impact. We construct an Input-Output Hidden Markov Model (IOHMM) denoted as M, which is a tuple $(H, E, \Psi, \Theta, n, \gamma)$.

- $n$ is a parameter for the number of discrete time points that M will be modeling.
- $H$ is a set of hidden states. Each hidden state, $h$, is associated with a Gaussian output distribution.
- $\Theta$ is the set of parameters for the Gaussian output distribution. For each hidden state h, $\Theta_h$ is a tuple $(\mu_h, \sigma_h)$ of the mean and standard variation of the Gaussian output distribution associated to h.
- $E$ is the set of directed edges connecting hidden stats of H, corresponding to valid transitions among hidden states.
- $\Psi$ is the set of transition parameters between hidden states. For each hidden state h, if a state $h \in H$ has two or more children, that is there are $a, b \in H$ such at (h,a) and (h,b) $\in E$ and $a \neq b$, there there is an element $\Psi_h \in \Psi$, $\Psi_h$ is a vector of parameters for a logistic regression classifier. To infer the transition model (logistic regression classifier), we need to utilize additional regulatory information such as epigenomic data and proteomics data.
- $\gamma$ is the maximal number of children for each node.

## Integrating proteomics and protein-protein interactions

While some methods further integrated these datasets with proteomics data (for example, SDREM [15]) to date these have been static interaction datasets. In contrast, in our study

we have also profiled a complementary time series proteomics data set that we integrated into the DREM model. While there is conflicting evidence about the correlation between mRNA and protein levels, for TFs specifically several studies indicate that expression levels are usually not enough to determine their activity levels. We have thus used the proteomics data to improve our ability to detect the *time* of TF activation. Specifically, we look for two lines of evidence to determine such activity. The first is the level of the protein itself and the second is the likelihood of a post-translational interaction or modification that leads to its activation. For the former we use the proteomics data directly. For the latter we combine protein-interaction data (known knowledge from previous studies, e.g. from public database such as STRING V10.5 used in the study) with the proteomics data as follows: We look at the average protein levels of its interacting partners and if these partners are expressed at a high level we increase our belief in the activity of the TF, even if the TF itself is not over expressed.

$$ATF_{Raw}(x,t) = \frac{1}{|Y|} \sum_{y \in |Y|} P(x,t)P(y,t)PPI(x,y) \tag{1}$$

$$ATF(x,t) = \frac{1 - e^{-w_{ATF} ATF_{Raw}(x,t)}}{1 + e^{-w_{ATF} ATF_{Raw}(x,t)}} \tag{2}$$

Where ATF(x,t) represents the inferred activity of TF $x$ at time $t$ based on the proteomics data, $Y$ denotes the set of proteins that interact with $x$ and $PPI(x,y)$ is the static protein-protein interaction strength between TF $x$ and $y$ obtained from STRING v10 database [16]. $P(x,t), P(y,t)$ represent the protein level for TF $x$ and interacting protein $y$ respectively. As we discuss below, this activity is then used as a *dynamic prior* by our model in order to better determine which TFs regulate which bifurcation event. Here, we used the shifted logistic function to normalize the Activity of TFs (ATF) to [0,1]. Please note that the range of the shifted logistic function described in eqn.(2) is [-1,1] in principle. However, all the expression profiles (including the proteomics data) are all non-negative ($\geq 0$). Therefore, the normalized value $ATF(x,t) \in [0,1]$. $w_{ATF}$ is a parameter which controls the steepness of the logistic function. $ATF(x,t)$ is a normalized quantification of the protein level of x at time point t. Besides, in practice, the proteomics data is always limited. In some extreme cases, we only have the protein level for a few hundred proteins. For those missing proteins, we are using an estimated protein level based on expression of corresponding genes. To make such estimation, we build a logistic function to project the expression level to protein level. We choose the logistic function weight, which minimizes the error between true protein level and the protein level predicted based on the gene expression data.

## Integrating epigenomic data

While we cannot usually obtain direct time series measurements for the binding of TFs (for example, time series ChIP-Seq data for all TFs, which requires several additional experiments for each time point) we can often obtain global indirect information about such events. For example, DNA methylation data was shown to correlate with other epigenomic datasets [17–19] and with binding for several TFs [20–22] and can be profiled globally. We can use DNA methylation data to obtain a prior on the dynamic binding events for different TFs. DNA methylation is often thought to prevent TF binding by changing the chromatin structure which restricts the access of TFs to the promoter regions [23]. We thus use the time series methylation data to identify "silenced" TFs. These are TFs that, while active, may seem to be inactive for some targets because their binding sites for these targets are methylated. In the original DREM method, such TFs would be assigned a low score (since several of their targets are inactive) and would thus be wrongly removed from the model. For this, we revise the static prior interaction map used by DREM (that assigns each TF-target pair a likelihood of being a target) and reduce this likelihood for genes with methylated promoters. This reduction places more weight on non-methylated targets when compared to methylated ones and so may allow a better identification of active TFs. Specifically, use the following as the methylation score:

$$Mr(y,t) = 1 - methyl(y,t) \tag{3}$$

Where $Mr(y,t)$ represents the regulation "score" for gene $y$ at time $t$ based on the given methylation data. $methyl(y,t)$ is the average methylation of the promoter of gene $y$ at time point $t$. Here $methyl(y,t)$ must be the normalized DNA methylation level in range [0,1]. If the methylation score is already in range [0,1] (e.g. Bisulfite-Seq score), then we don't need to normalize the methylation score. If not, we will have to normalize the methylation score to [0,1]. Note, the methylation scores must be pre-processed. In other words, the methylation input for the iDREM model must be already normalized to range [0,1]. Please refer to iDREM manual for normalization instructions.

The DNA methylation data is not the only data can be used to infer the TF binding prior. The other epigenomic data such as Histone modification can also be used to infer the TF binding prior. For example, H3K4me2/3 are generally associated with transcriptional activity and H3K27me2/3 are associated with repression [24]. With this information, we can calculate $Mr(y,t)$ similarly as the DNA methylation data.

$$Mr(y,t) = \begin{cases} Epi_{score}(y,t) \ , if\ transcriptional\ activity \\ 1 - Epi_{score}(y,t) \ , if\ repression \end{cases} \tag{4}$$

$Epi_{score}(y,t)$ represents the epigenomic score of y at time t. The "activation" and "repression" associated scores are treated differently in iDREM. By default, the iDREM takes the "repression" epigenomic scores (such as DNA methylation score), if an "activation" epigenomic score is provided (e.g. H3K4me2), please transform it to "repression" before using it as the iDREM input. $(x- > 1 - x)$. Please refer to the manual for more details.

## Inferring dynamic input TF-DNA regulation map

For integration of other data (e.g. miRNA), which was already incorporated in the previous DREM, please refer to [4]. We now explain how the different dynamic data sources are combined to derive a dynamic prior for the regulation of a gene by a TF. This dynamic prior is then combined with the dynamic gene expression information of the target gene in order to group genes in paths and infer TF activity. For the dynamic prior we combined the TF activity value ($ATF(x,t)$) derived from the time series proteomics data, the methylation score $Mr(y,t)$ and a static TF-DNA prior $R_{static}(x,y)$. The way these are combined is as follows:

$$R(x,y,t) = R_d(x,y,t)R_v(x,y,t) \tag{5}$$

$$R_v(x,y,t) = \frac{1 - e^{-w_R|\Delta ATF(x,t)|TBS(x,y,t)}}{1 + e^{-w_R|\Delta ATF(x,t)|TBS(x,y,t)}} \tag{6}$$

$$R_d(x,y,t) = \begin{cases} -1, \ if \Delta ATF(x,t)\Delta Ex(y,t) < 0 \\ 1, \ if \Delta ATF(x,t)\Delta Ex(y,t) > 0 \\ 0, \ else \end{cases} \tag{7}$$

$$\Delta ATF(x,t) = ATF(x,t) - ATF(x,t-1) \tag{8}$$

$$\Delta Ex(y,t) = Ex(y,t) - Ex(y,t-1) \tag{9}$$

$$TBS(x,y,t) = Mr(y,t)R_{static}(x,y) \tag{10}$$

Where $R(x,y,t)$ is the dynamic prior for the regulation by TF $x$ of target gene $y$ at time point $t$. $R_d(x,y,t)$ represents the Regulation direction (activation or repression) and $R_v(x,y,t)$ represents the regulation strength between TF $x$ and target gene $y$ at time $t$. $Ex(y,t)$ represents the expression of $y$ at time point $t$. $ATF(x,t)$ denotes the estimated TF 'activity' based on proteomics and PPI data. $R_{static}(x,y)$ denotes the strength of binding of

TF $x$ on gene $y$ based the static TF-DNA regulation used in the study. $Mr(y,t)$ represents the TF regulation 'score' for gene $y$ at time point $t$ based on the methylation data. Basically we first combine the two terms that are based on DNA studies (in the $TBS$ terms which combines the methylation and ChIP-Seq or sequence information) and then combine that with the activity value. The value derived for $R(x,y,t)$ is then used by DREM as we discuss below. Here, we used a shifted logistic function for normalization. $R_v(x,y,t)$ is a normalized TF binding prior in the range [0,1]. $w_R$ is a logistic function weight parameter used for the normalization.

we have three types of data that are *directly* related to such activity: Information regarding TF-gene and miRNA-gene interaction (usually collected from several other experimental studies and so not necessarily relevant to the specific model, so they serve as a prior), miRNA expression levels (indicating the activity level of miRNAs) and information about the target gene expression which is the direct consequence of such interaction. These three information sources are the core information used by iDREM and they are the most influential. For these direct sources we do not normalize all modalities to [0,1]. Specifically, for the mRNA and miRNA expression we use the actual values (log2 fold change to time point 0) both when learning the logistic regression function for each split and for the final model we display. Higher values will lead to higher impact and vice versa. In addition, for these information sources it is hard to determine an exact weight as they are used differently in the logistic regression method that iDREM employs to infer TF / miRNA activity (expression as the output and interactions as the inputs). Note that for this data iDREM allows users to upload their own (prior) interaction matrix and so users can assign higher weights to specific interactions they know about.

The others are indirect information sources (protein / expression level of the TF which is a good indication but not definitive since the TF can be stable in terms of expression but still post-transcriptionally activated and methylation information). These latter information sources indeed require weighting since they are used to influence the prior on the activity of the individual TFs or their targets. We thus both normalize them to [0,1] and assign equal weight to both (basically the final prior is adjusted by a function that is a product of both values, see Eq. 5-10 in the supplement for details). Without any prior knowledge, here we assume the equal weights for proteomics and epigenomics data. However, uses are able to change the relative importance of the proteomics and epigenomics data. Please refer to iDREM manual (https://github.com/phoenixding/idrem) fore details.

## Likelihood density function

By combining the inputs from all datasets, we get a dynamic input vector $I(g,t)$ for each gene g at each time point t. $I(g,t)$ is composed by two parts: TF regulation vector and miRNA

regulator vector. The TF-DNA regulation map was inferred using the dynamic proteomics, dynamic methylation, static PPI, static TF-DNA and dynamic gene expression data as we discussed in the above section. $R(x, y, t)$ is used to represent the dynamic TF-regulation between TF $x$ and gene $y$ at time point t. The miRNA-mRNA regulation map is inferred using the same strategy as described in our previous work mirDREM [14], here we used $R_m(x, y, t)$ to represent the dynamic miRNA regulation between miRNA $x$ and target gene $y$ at time point $t$. The overall input vector for gene $g$ is:

$$I_{g,t} = [R(x_0, g, t), ..., R_(x_i, g, t), ..., R_(x_p, g, t), R_m(y_0, g, t), ..., R_m(y_j, g, t), ..., R_m(y_q, g, t)] \quad 0 \leq i \leq p, 0 \leq j \leq q \tag{11}$$

Where $x_0, ..., x_p$ denotes all TF binding to $g$ and $y_0, ..., y_q$ represents all miRNAs binding to $g$. Note, for miRNA regulation $R(x_p, g, t)$, we only allow repressing effect, which is indicated by the difference between $R(x, y, t)$ and $R_m y(x, y, t)$ calculation. Please refer to supplements for the complete details.

Let $O_g = (O_g(1), O_g(2), ..., O_g(n-1))$ be the log ration expression values for gene g at time points 1 to n-1 to a time point 0 control and $h_t$ be the hidden state at time t ($t \in 0, 1, ..., n-1$). The probability of transition of gene $g$ from state $h_a$ at time t-1 to state $h_b$ at t is defined as $P(H_t = h_b | H_{t-1} = h_a, I(g, t))$. This probability is defined as 0 if $h_b$ is not a child of $h_a$ and 1 if $h_b$ is the only child of $h_a$. If $h_a$ has two or more children, then the transitions are probabilistic and depend on the input vector $I(g, t)$, which were determined by a logistic regressor discussed in previous DREM studies [4,13,14]. The likelihood density, r, for a set of genes G in IOHMM model M is :

$$r(G|M) = \sum_{g \in G} log \sum_{q \in Q} \prod_{t=1}^{n-1} f_{q(t)}(o_g(t)) \prod_{t=1}^{n-1} P(H_t = q(t) | H_{t-1} = q(t-1), I(g, t)) \tag{12}$$

Where $f_{q(t)}(o_g(t))$ represents the emission probability from state $q(t)$ to observation $o_g(t)$. Q is the set of all paths of hidden stats of length n starting from the root. For a path $q \in Q$, $q(t)$ is the hidden state of the path at time point t. The first product denotes the emission probability and the second product represents the transition probability. The inner sum is over all paths and the outer sum is over all genes in G. $I(g, t)$ is the dynamic input prior learned by integrating all different types of data as we discussed above.

## Model learning

As the model M is defined as a tuple M=$(H, E, \Psi, \Theta, n, \gamma)$. $n$ and $\gamma$ are 2 pre-defined parameters. $H, E$ represent the model structure. $\Psi, \Theta$ represent the model parameters.

To learn the parameters ( $\Psi, \Theta$), we used the Baum-Welch algorithm on a given structure denoted by current $H, E$. During each maximization step of Baum-Welch, the logistic regression classifiers are retrained. When training a classifier, for every gene in the training set the classifier is given a weighted example of the gene transition to each child state. The weight of the example is the probability of that gene going through that hidden state based on the current values of all the parameters in the model.

The structure $(H, E)$ is learned in a greedy fashion. Starting from a linear model with one hidden state per time point, the algorithm explores additions of new hidden states at each time point. The algorithm uses the BIC penalty to the model likelihood to decide if the best hidden state addition is executed and the model structure is changed, in each round of structure learning. A new state is added if the penalized likelihood is larger than in the unchanged model (default penalty 40). After no new addition is made at the end of a structure learning round, it is tested if splits can be delayed or paths merged to simplify the model. After structure learning, all genes are parsed through the model and assigned to paths in the model using the Viterbi algorithm.

# Supporting Tables

Supporting Table 1: mouse microglia development time points used in this paper

| data | reference ID | Data type | time points | | | | | | | | | |
|---|---|---|---|---|---|---|---|---|---|---|---|---|
| mRNA expression data | PMID27338705 | Microglia | E10.5 | E11.5 | E12.5 | E13.5 | E14.5 | E16.5 | P3 | P6 | P9 | Adult |
| histone methylation data | PMID27338705 | Microglia | - | - | E12.5 | - | E14.5 | E16.5 | P3 | - | - | Adult |
| miRNA expression | PMID15345052 | whole brain | - | - | E12.5 | - | - | E16.5(E17.5) | P3(P4) | - | - | Adult |
| proteomics data | PMID18283662 | whole brain | E10.5 (E9.5) | E11.5 | - | E13.5 | - | - | - | - | - | - |

Supporting Table 2: Top GO terms for each path

| Path | Top 3 GO terms |
|---|---|
| A | cell-cell adhesion via plasma-membrane adhesion molecules (p-value:2.96e-12; Bonferroni corrected p-value:3.46e-08)<br>homophilic cell adhesion via plasma membrane adhesion molecules(p-value:3.09e-12;Bonferroni corrected p-value:3.62e-08)<br>nervous system development (p-value:4.70e-10; Bonferroni corrected p-value:5.51e-6) |
| B | regulation of cell communication(p-value:2.59e-5; Bonferroni corrected p-value:0.304)<br>regulation of signaling(p-value:2.70e-5; Bonferroni corrected p-value:0.317)<br>regulation of response to stimulus(p-value:4.67e-5; Bonferroni corrected p-value:0.547) |
| C | intracellular (p-value:8.27e-15; Bonferroni corrected p-value:9.68e-11)<br>cellular metabolic process (p-value:4.62e-11; Bonferroni corrected p-value:5.41e-7)<br>macromolecule biosynthetic process (p-value:4.89e-11; Bonferroni corrected p-value:5.73e-7) |
| D | cytosolic ribosome (p-value:3.92e-34; Bonferroni corrected p-value:4.59e-30)<br>structural constituent of ribosome (p-value:6.66e-30; Bonferroni corrected p-value:7.80e-26)<br>ribosomal subunit (p-value:7.15e-30; Bonferroni corrected p-value:8.37e-26) |
| E | nuclear nucleosome (p-value:3.99e-7; Bonferroni corrected p-value:4.67e-3)<br>DNA packaging complex(p-value:1.56e-6; Bonferroni corrected p-value:0.0183)<br>immune response (p-value:2.15e-6; Bonferroni corrected p-value:0.0252) |
| F | nervous system development (p-value:8.61e-33; Bonferroni corrected p-value:1.01e-28)<br>system development (p-value:9.89e-32; Bonferroni corrected p-value:1.16e-27)<br>anatomical structure development (p-value:1.31e-28; Bonferroni corrected p-value:1.54e-24) |
| G | intracellular membrane-bounded organelle (p-value:1.17e-44; Bonferroni corrected p-value:1.37e-40)<br>intracellular part(p-value:5.58e-44; Bonferroni corrected p-value:6.54e-40)<br>membrane-bounded organelle (p-value:3.10e-43; Bonferroni corrected p-value:3.64e-39) |
| H | Cytoplasm (p-value:5.54e-9; Bonferroni corrected p-value:6.50e-5)<br>cytoplasmic part (p-value:2.20e-8; Bonferroni corrected p-value:2.58e-4)<br>protein binding (p-value:5.67e-8; Bonferroni corrected p-value:6.64e-4) |

Supporting Table 3: supported regulating factors predicted by iDREM

| TF | p-value | reported regulating stages | predicted regulating stages by iDREM |
|---|---|---|---|
| Rxrb | - | E12.5-Adult | - |
| Fli1 | 2.95e-47 | E14-Adult | E12.5- Adult |
| Egr1 | 4.23e-58 | E14-Adult | E14.5-Adult |
| Fos | 1.24e-58 | E14-Adult | E12.5-Adult |
| Mafb | - | Adult | - |
| Mef2a | 2.06e-48 | E16.5, P3 , Adult | E13.5-Adult |
| Jun | 1.74E-67 | E12.5, E14, Adult | E12.5-Adult |

Supporting Table 4: regulator comparison for models using different sets of input data

| Regulator | model I | model II | model III | model IV |
|---|---|---|---|---|
| Rxrb | No | No | No | No |
| Fli1 | No | No | Yes | Yes |
| Egr1 | No | No | No | Yes |
| Fos | Yes | Yes | Yes | Yes |
| Mafb | No | No | No | No |
| Mef2a | Yes | Yes | Yes | Yes |
| Jun | Yes | Yes | Yes | Yes |
| Cd40 | Yes | Yes | Yes | Yes |
| Smad1 | Yes | Yes | Yes | Yes |
| Traf4 | No | No | No | Yes |
| number of predicted verified TFs | 5 | 5 | 6 | 8 |

# Supporting Figures

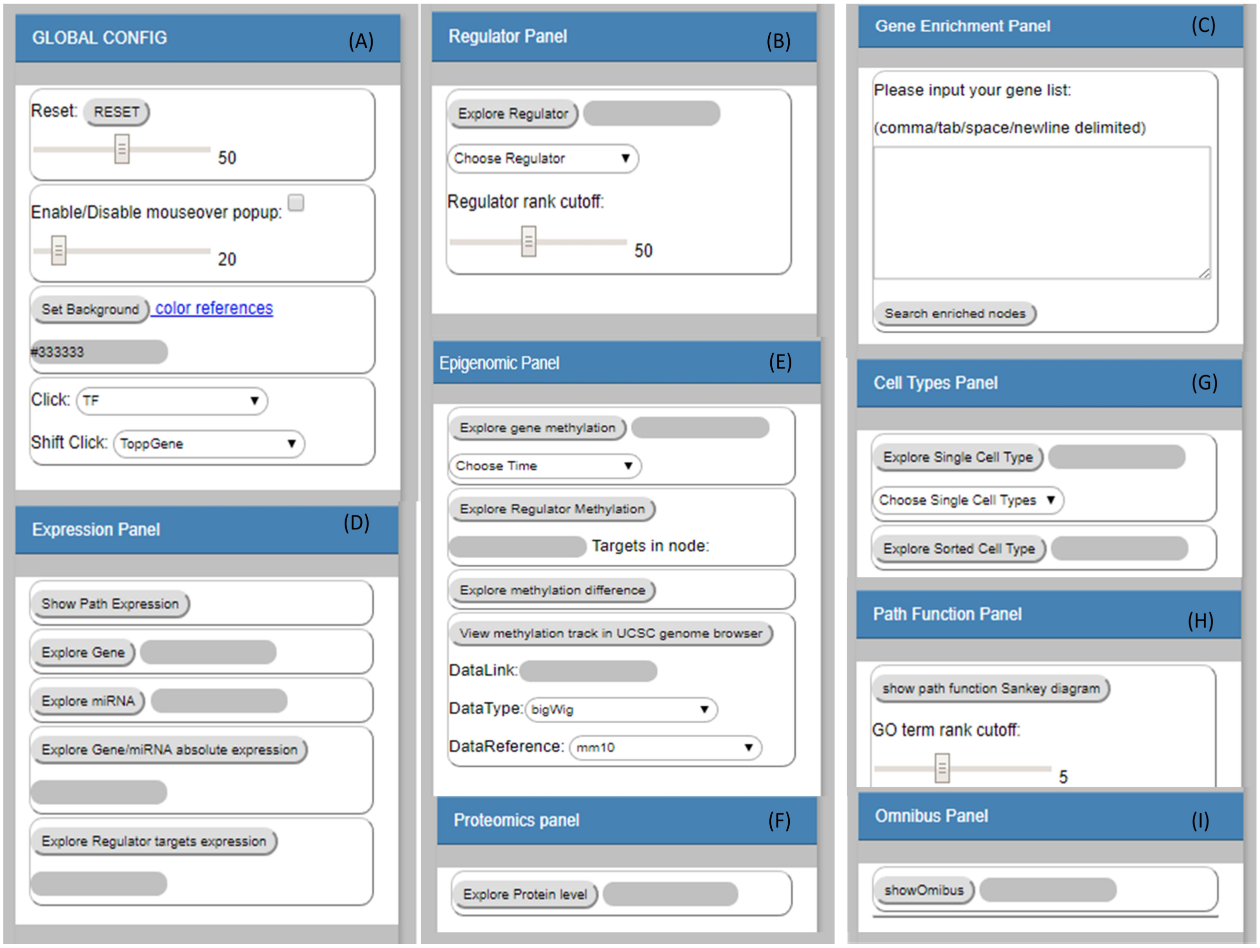


Supporting Figure 1: **iDREM visualization configuration panels. See text for detailed explanation of each.**

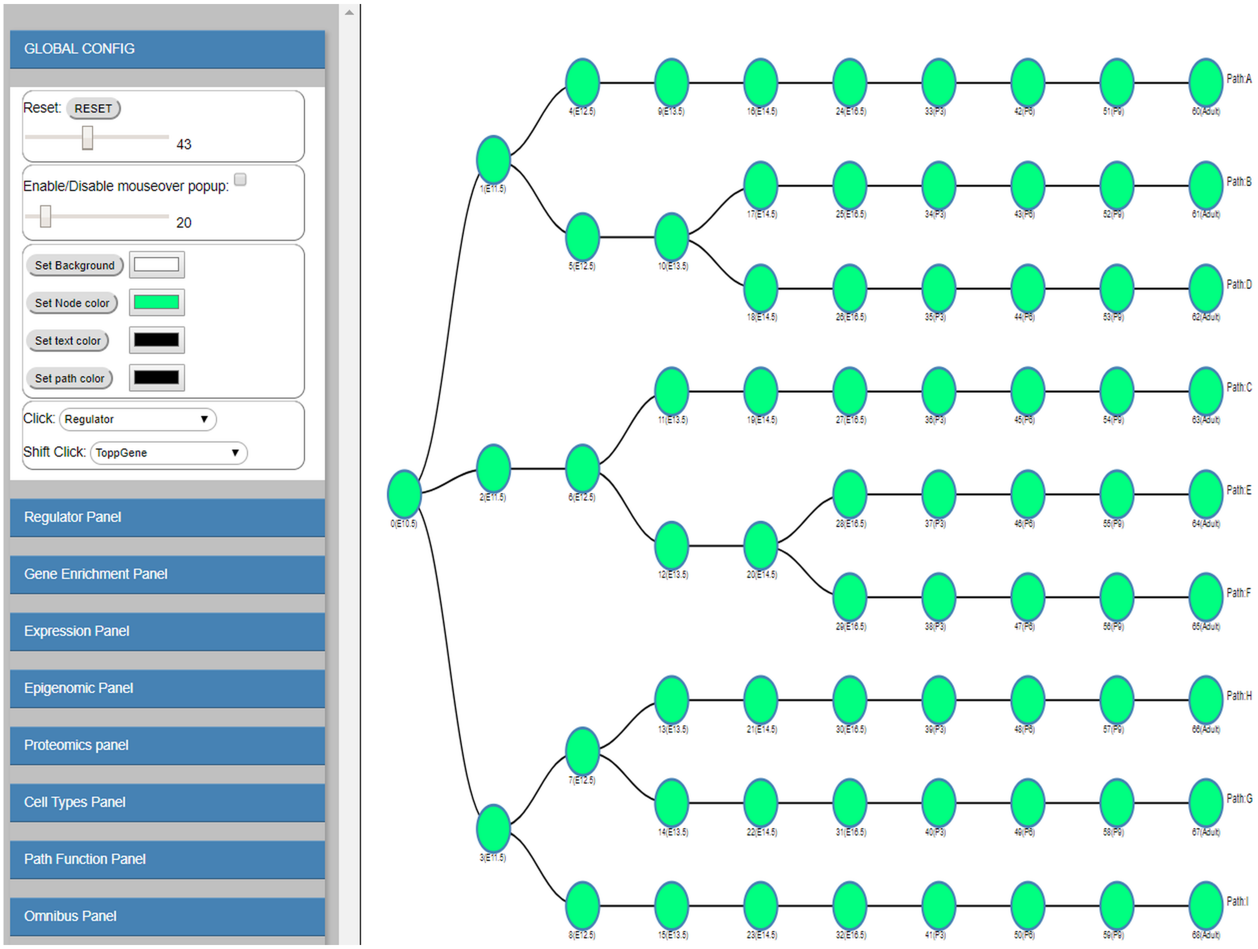


Supporting Figure 2: **iDREM interactive visualization**

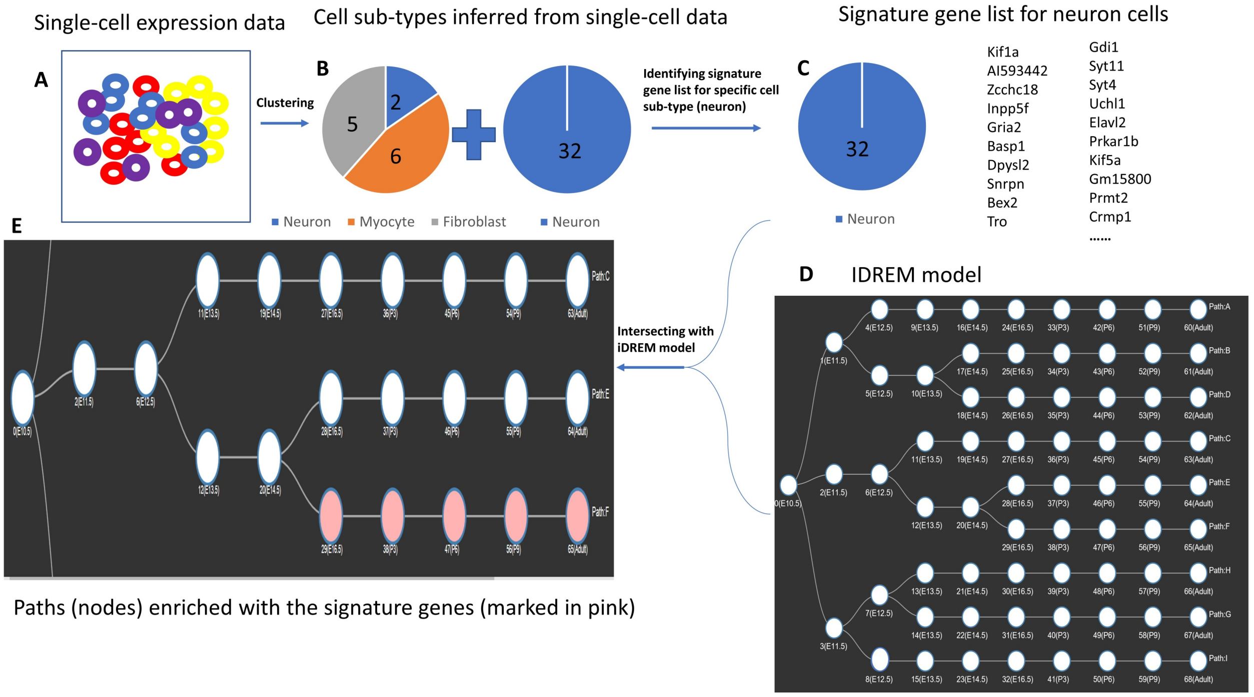


Supporting Figure 3: **An usage example of single-cell RNA-seq data**

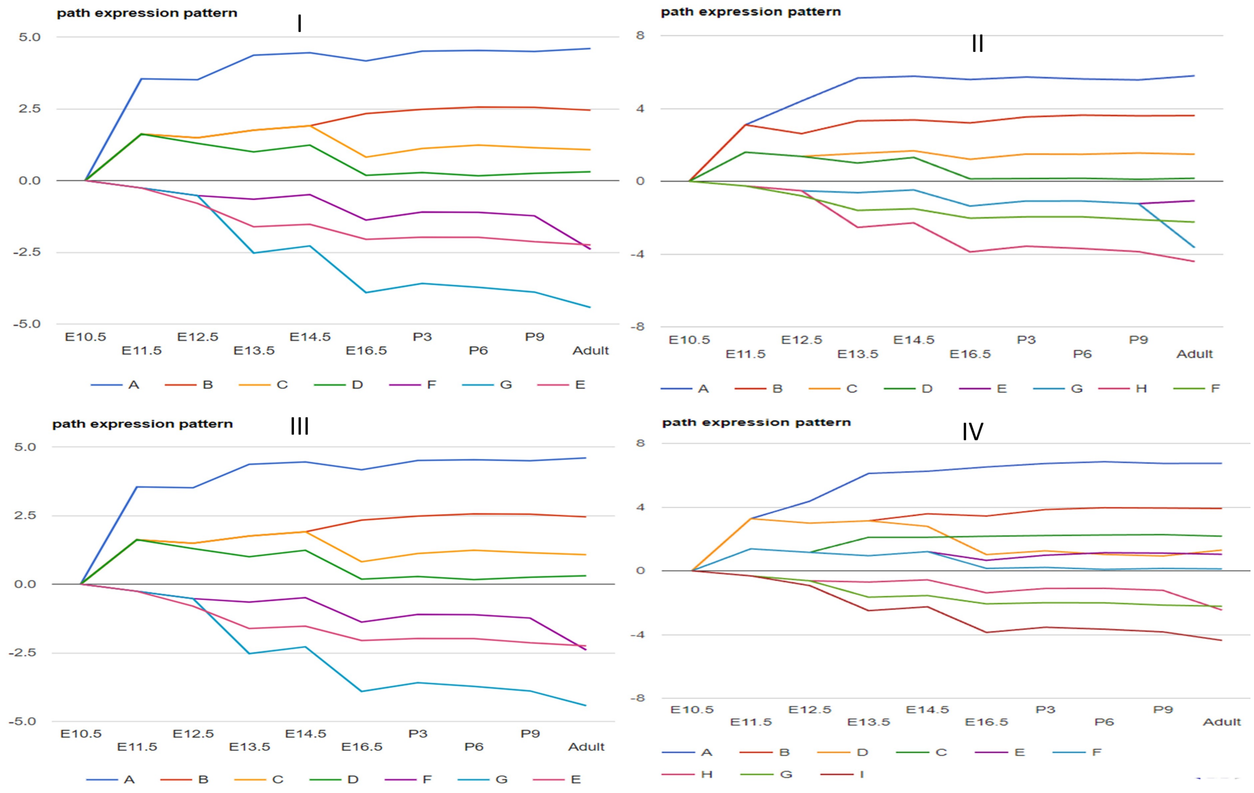


Supporting Figure 4: **predicted paths for model: I, II, III, IV**. I: only use miRNA and mRNA expression data; II: data used by I+ time series proteomics data; III: the data used by I + the time series methylation data; IV: using all data presented in this study.

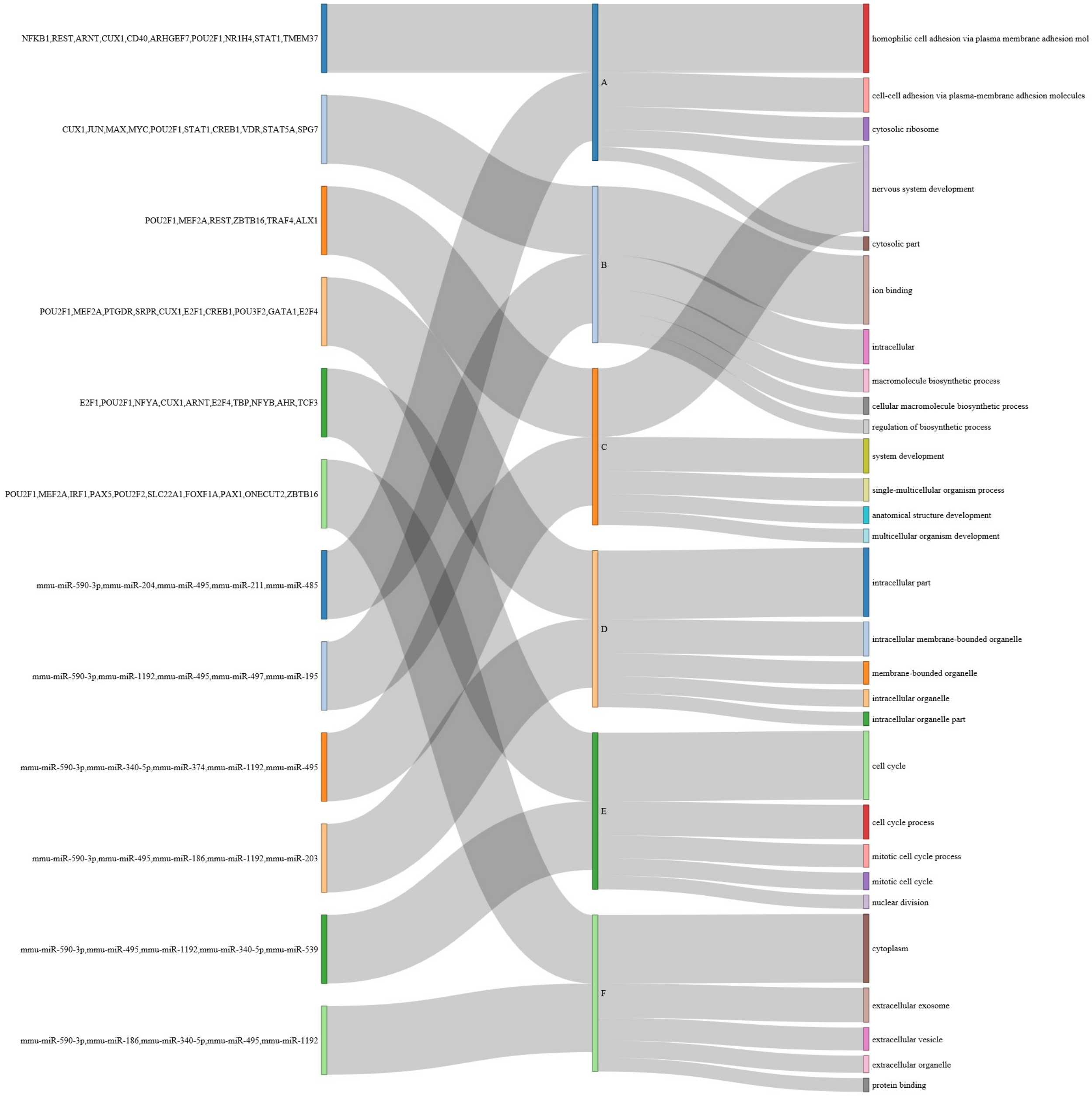


Supporting Figure 5: **Sankey Diagram for model I**. In this diagram, the path GO functions and regulators are shown.

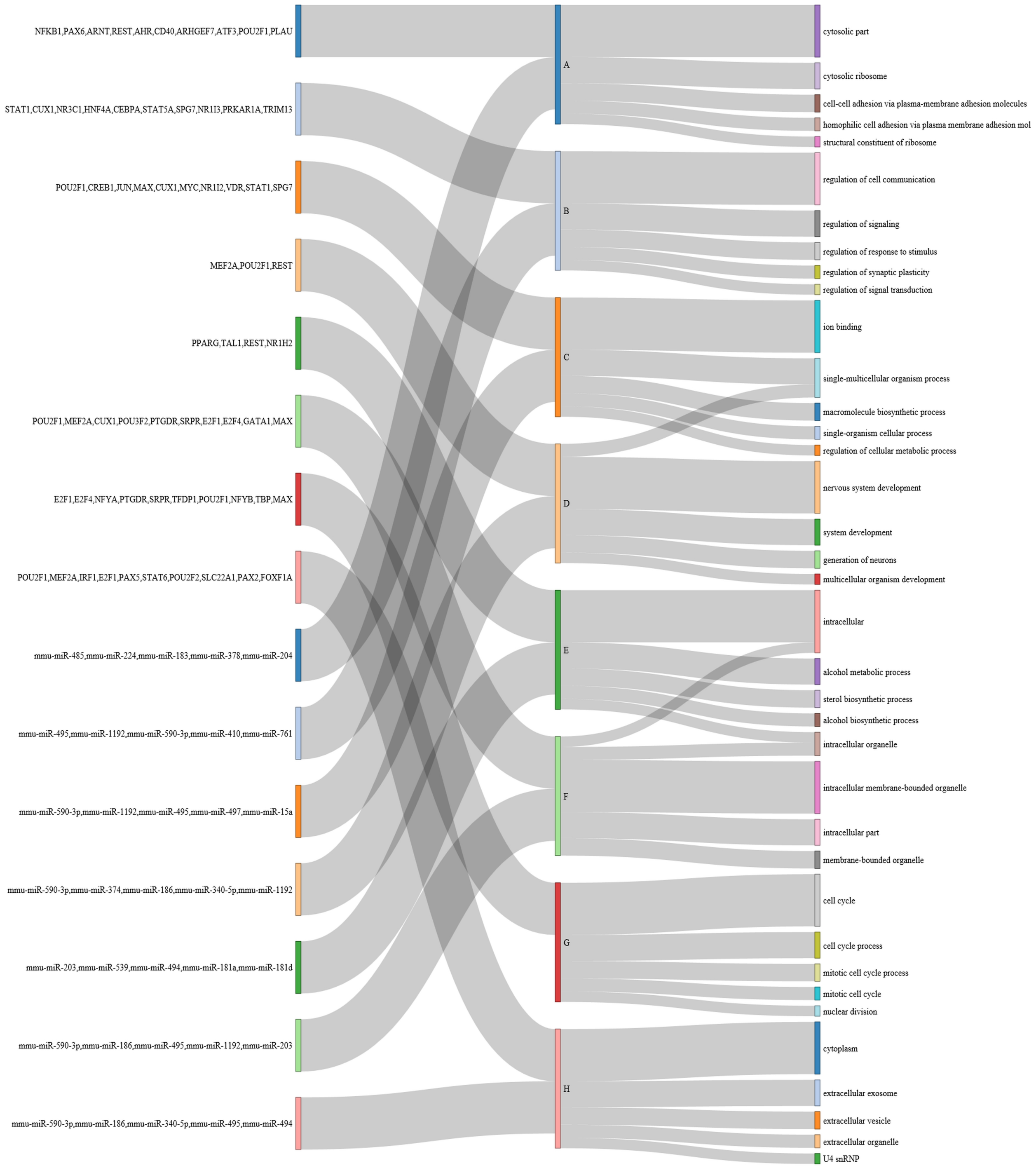


Supporting Figure 6: **Sankey Diagram for model II**. In this diagram, the path GO functions and regulators are shown.

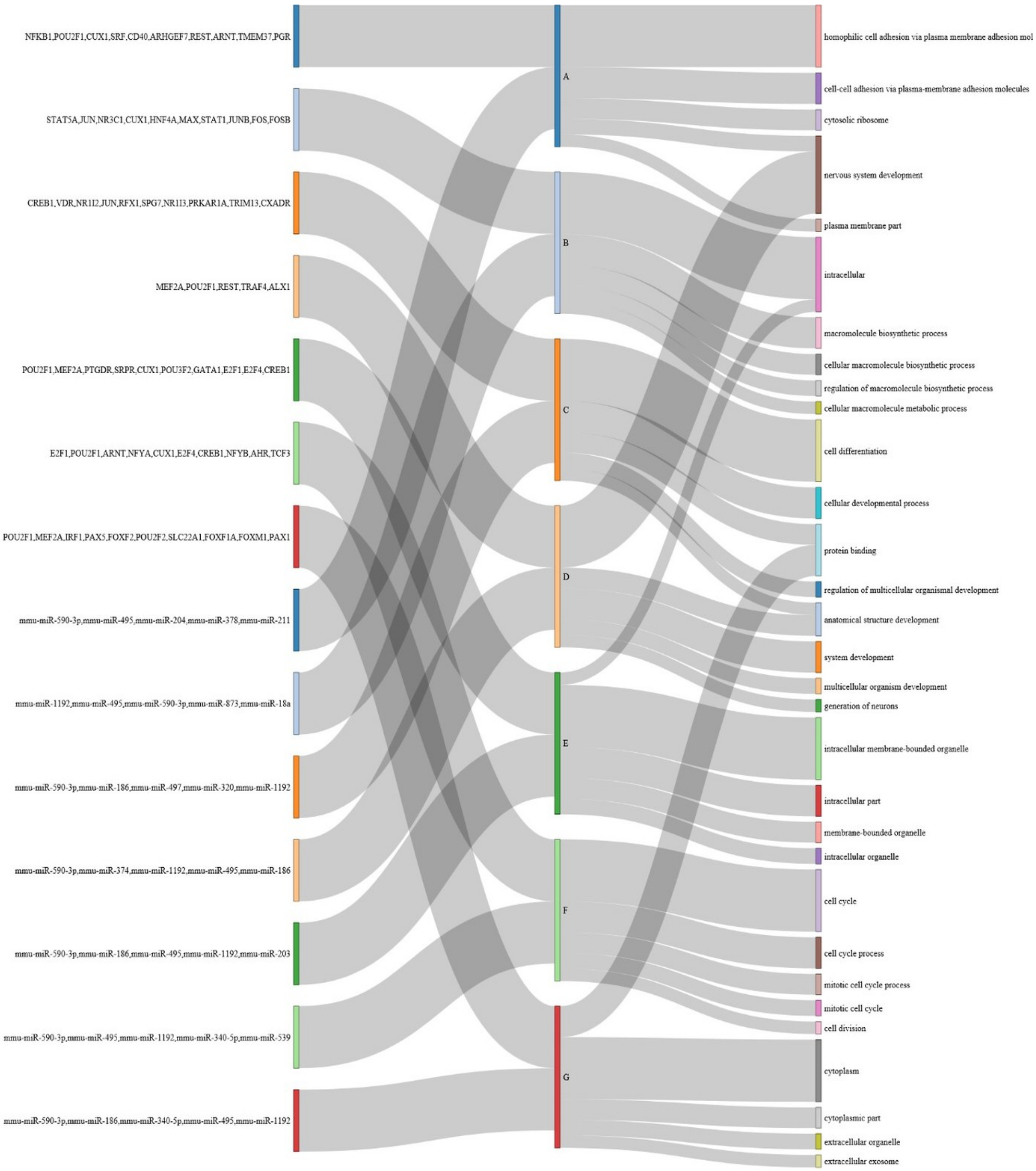


Supporting Figure 7: **Sankey Diagram for model III**. In this diagram, the path GO functions and regulators are shown.

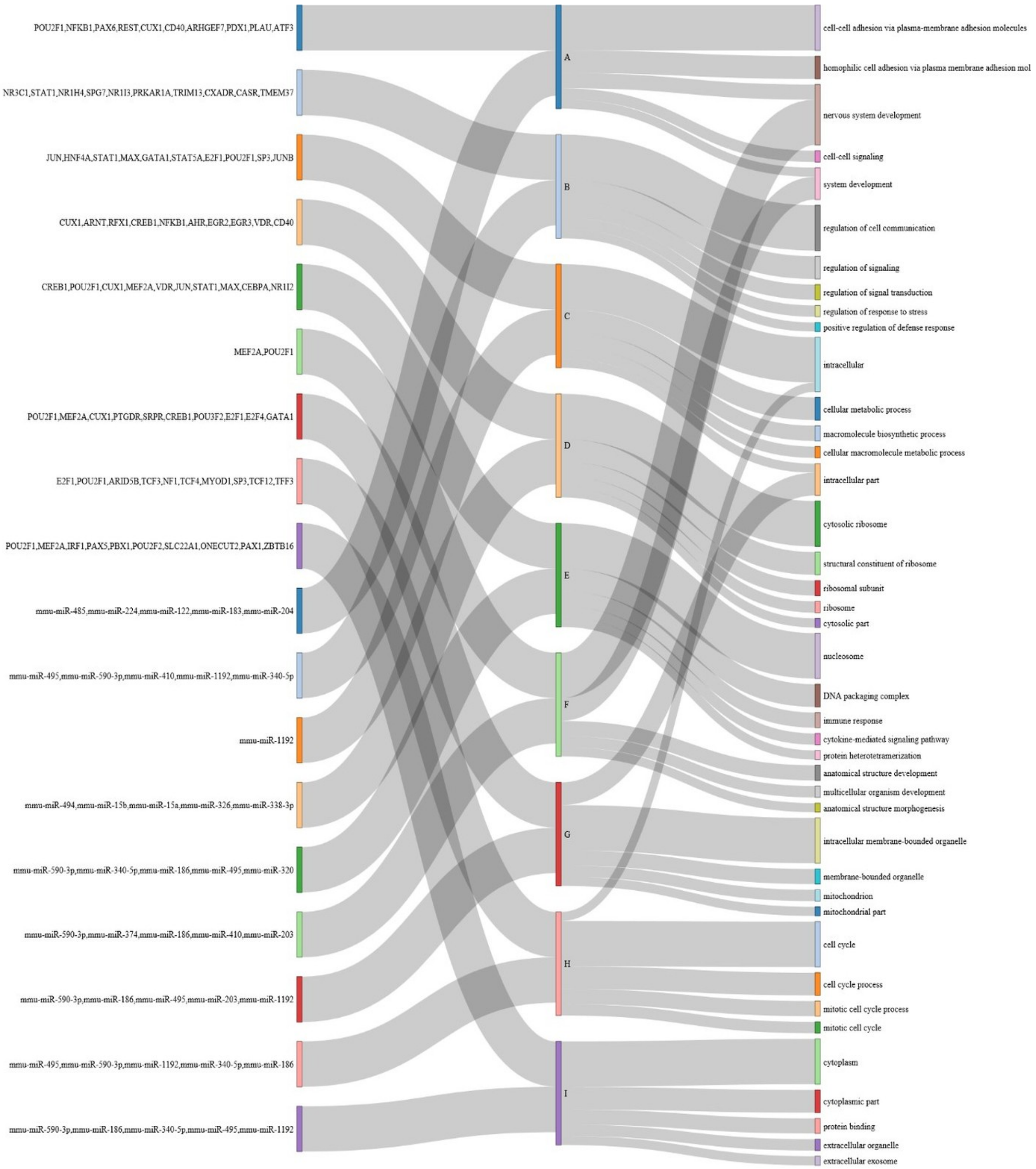


Supporting Figure 8: **Sankey Diagram for model IV**. In this diagram, the path GO functions and regulators are shown.

# References


[1] Pugliatti L, Derre J, Berger R, Ucla C, Reith W, Mach B. The genes for MHC class II regulatory factors RFX1 and RFX2 are located on the short arm of chromosome 19. Genomics. 1992;13(4):1307–1310.

[2] Streit WJ, Conde JR, Fendrick SE, Flanary BE, Mariani CL. Role of microglia in the central nervous system's immune response. Neurological research. 2005;27(7):685–691.

[3] Karolchik D, Baertsch R, Diekhans M, Furey TS, Hinrichs A, Lu Y, et al. The UCSC genome browser database. Nucleic acids research. 2003;31(1):51–54.

[4] Schulz MH, Devanny WE, Gitter A, Zhong S, Ernst J, Bar-Joseph Z. DREM 2.0: Improved reconstruction of dynamic regulatory networks from time-series expression data. BMC systems biology. 2012;6(1):104.

[5] Abbas AR, Baldwin D, Ma Y, Ouyang W, Gurney A, Martin F, et al. Immune response in silico (IRIS): immune-specific genes identified from a compendium of microarray expression data. Genes and immunity. 2005;6(4):319.

[6] Treutlein B, Brownfield DG, Wu AR, Neff NF, Mantalas GL, Espinoza FH, et al. Reconstructing lineage hierarchies of the distal lung epithelium using single-cell RNA-seq. Nature. 2014;509(7500):371–375.

[7] Eramo A, Lotti F, Sette G, Pilozzi E, Biffoni M, Di Virgilio A, et al. Identification and expansion of the tumorigenic lung cancer stem cell population. Cell death and differentiation. 2008;15(3):504.

[8] Du Y, Kitzmiller JA, Sridharan A, Perl AK, Bridges JP, Misra RS, et al. Lung Gene Expression Analysis (LGEA): an integrative web portal for comprehensive gene expression data analysis in lung development. Thorax. 2017; p. thoraxjnl–2016.

[9] Matcovitch-Natan O, Winter DR, Giladi A, Aguilar SV, Spinrad A, Sarrazin S, et al. Microglia development follows a stepwise program to regulate brain homeostasis. Science. 2016;353(6301):aad8670.

[10] Chen J, Bardes EE, Aronow BJ, Jegga AG. ToppGene Suite for gene list enrichment analysis and candidate gene prioritization. Nucleic acids research. 2009;37(suppl_2):W305–W311.

[11] Consortium GO, et al. Gene ontology consortium: going forward. Nucleic acids research. 2015;43(D1):D1049–D1056.

[12] Sharma P, Schiapparelli L, Cline HT. Exosomes function in cell–cell communication during brain circuit development. Current opinion in neurobiology. 2013;23(6):997–1004.

[13] Ernst J, Vainas O, Harbison CT, Simon I, Bar-Joseph Z. Reconstructing dynamic regulatory maps. Molecular systems biology. 2007;3(1):74.

[14] Schulz MH, Pandit KV, Cardenas CLL, Ambalavanan N, Kaminski N, Bar-Joseph Z. Reconstructing dynamic microRNA-regulated interaction networks. Proceedings of the National Academy of Sciences. 2013;110(39):15686–15691.

[15] Gitter A, Carmi M, Barkai N, Bar-Joseph Z. Linking the signaling cascades and dynamic regulatory networks controlling stress responses. Genome research. 2013;23(2):365–376.

[16] Szklarczyk D, Franceschini A, Wyder S, Forslund K, Heller D, Huerta-Cepas J, et al. STRING v10: protein–protein interaction networks, integrated over the tree of life. Nucleic acids research. 2014;43(D1):D447–D452.

[17] Jones PA, Takai D. The role of DNA methylation in mammalian epigenetics. Science. 2001;293(5532):1068–1070.

[18] Johnson LM, Cao X, Jacobsen SE. Interplay between two epigenetic marks: DNA methylation and histone H3 lysine 9 methylation. Current Biology. 2002;12(16):1360–1367.

[19] Li X, Wang X, He K, Ma Y, Su N, He H, et al. High-resolution mapping of epigenetic modifications of the rice genome uncovers interplay between DNA methylation, histone methylation, and gene expression. The Plant Cell. 2008;20(2):259–276.

[20] Zilberman D, Gehring M, Tran RK, Ballinger T, Henikoff S. Genome-wide analysis of Arabidopsis thaliana DNA methylation uncovers an interdependence between methylation and transcription. Nature genetics. 2007;39(1):61–69.

[21] Watt F, Molloy PL. Cytosine methylation prevents binding to DNA of a HeLa cell transcription factor required for optimal expression of the adenovirus major late promoter. Genes & development. 1988;2(9):1136–1143.

[22] Tate PH, Bird AP. Effects of DNA methylation on DNA-binding proteins and gene expression. Current opinion in genetics & development. 1993;3(2):226–231.

[23] Lim DH, Maher ER. DNA methylation: a form of epigenetic control of gene expression. The Obstetrician & Gynaecologist. 2010;12(1):37–42.

[24] Rice JC, Briggs SD, Ueberheide B, Barber CM, Shabanowitz J, Hunt DF, et al. Histone methyltransferases direct different degrees of methylation to define distinct chromatin domains. Molecular cell. 2003;12(6):1591–1598.
