# Supplementary material for: iDREM: Interactive visualization of dynamic regulatory networks
Source: PLoS Comput Biol. 2018 Mar 14;14(3):e1006019. doi: 10.1371/journal.pcbi.1006019 (PMC5868853; doi:10.1371/journal.pcbi.1006019)
Supplement: S1 Fig — (A) Global config, which can be used to customize the visualizations (e.g. background color, node color, visualization size). (B)Regulator Panel, which can be used to visualize the gene/miRNA expression. (C) Enrichment panel, which an be used to find the enriched paths/nodes in iDREM model for any given inputs. (D)Expression panel, which can be used to visualize gene or miRNA expression. (E) Epigenomics Panel, which can be used to explore and visualize the epigenomics data used in the study. (F) Proteomics Panel, which can be used to visualize the protein levels. (G) Cell Types Panel, which can be used to explore/visualize the Single-cell or Sorted-Cell data. (H) Path Function Panel, which can be used to visualize the associated GO functions and regulators for each path. (I) Omnibus Panel, which can be used to explore and visualize the TF/gene in all possible panels. For a more detailed description, please refer to iDREM manual. (PDF) [file pcbi.1006019.s002.pdf]

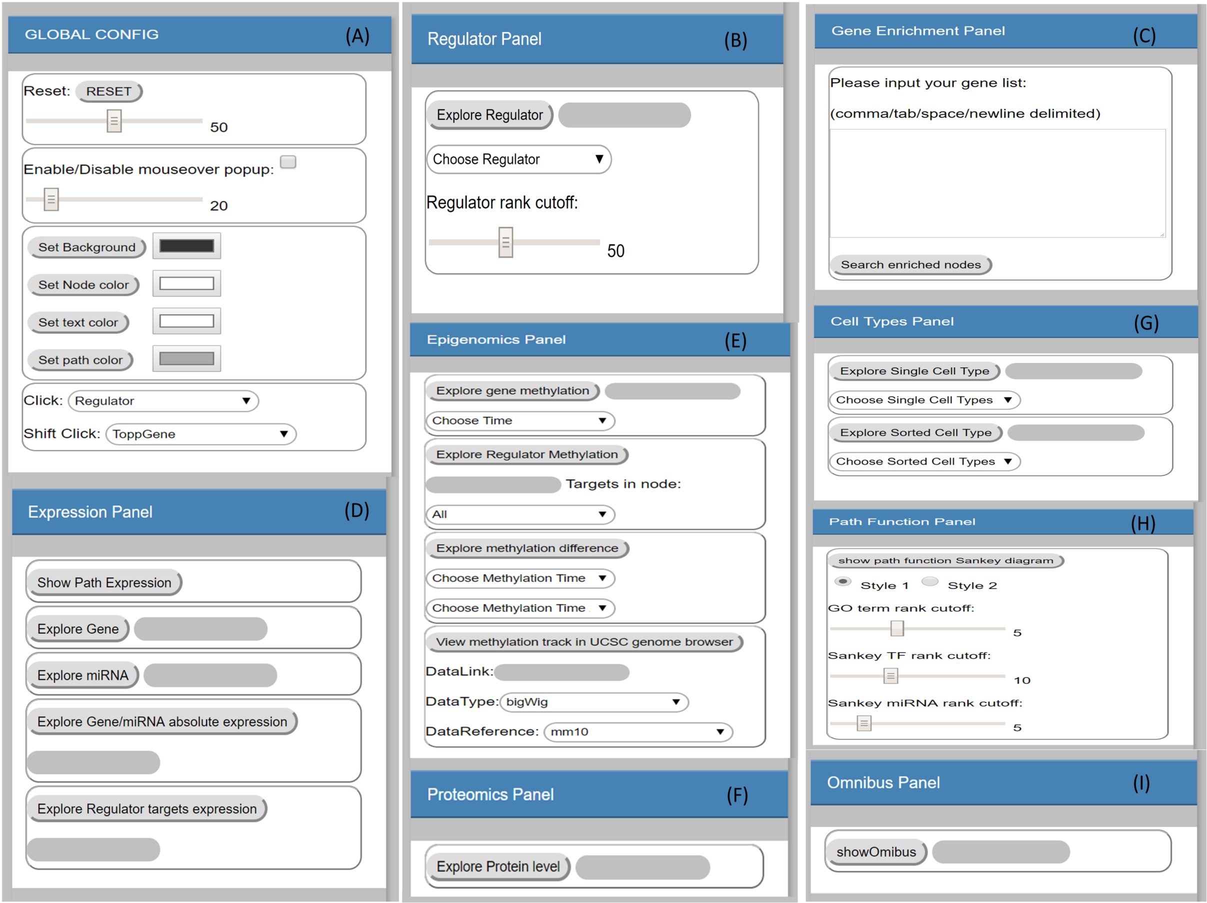
GLOBAL CONFIG
(A)
Reset: RESET
50
Enable/Disable mouseover popup:
20
Set Background
Set Node color
Set text color
Set path color
Click: Regulator
Shift Click: ToppGene
Expression Panel
(D)
Show Path Expression
Explore Gene
Explore miRNA
Explore Gene/miRNA absolute expression
Explore Regulator targets expression
Regulator Panel
(B)
Explore Regulator
Choose Regulator
Regulator rank cutoff:
50
Epigenomics Panel
(E)
Explore gene methylation
Choose Time
Explore Regulator Methylation
Targets in node:
All
Explore methylation difference
Choose Methylation Time
Choose Methylation Time
View methylation track in UCSC genome browser
DataLink:
DataType: bigWig
DataReference: mm10
Proteomics Panel
(F)
Explore Protein level
Gene Enrichment Panel
(C)
Please input your gene list:
(comma/tab/space/newline delimited)
Search enriched nodes
Cell Types Panel
(G)
Explore Single Cell Type
Choose Single Cell Types
Explore Sorted Cell Type
Choose Sorted Cell Types
Path Function Panel
(H)
show path function Sankey diagram
Style 1
Style 2
GO term rank cutoff:
5
Sankey TF rank cutoff:
10
Sankey miRNA rank cutoff:
5
Omnibus Panel
(I)
showOmnibus
