# Supplementary material for: iDREM: Interactive visualization of dynamic regulatory networks
Source: PLoS Comput Biol. 2018 Mar 14;14(3):e1006019. doi: 10.1371/journal.pcbi.1006019 (PMC5868853; doi:10.1371/journal.pcbi.1006019)

GLOBAL CONFIG

Reset: RESET

43

Enable/Disable mouseover popup:

20

Set Background

Set Node color

Set text color

Set path color

Click: Regulator

Shift Click: ToppGene

Regulator Panel

Gene Enrichment Panel

Expression Panel

Epigenomic Panel

Proteomics panel

Cell Types Panel

Path Function Panel

Omnibus Panel

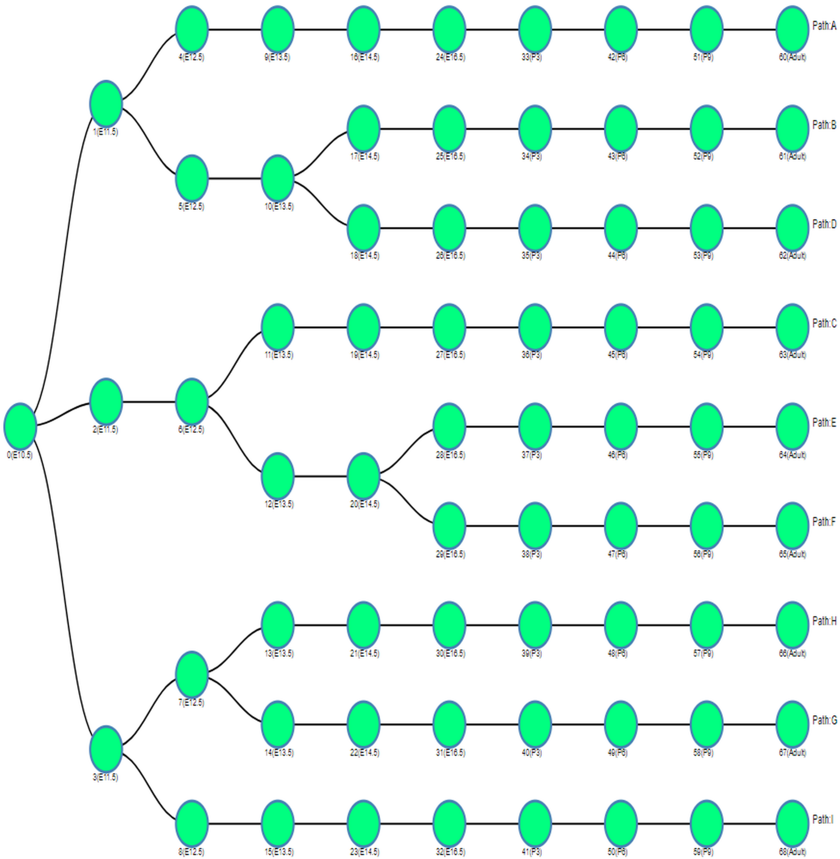

Supplement: S2 Fig — This figure shows the interactive visualization for the microglia data used in the study. (PDF) [file pcbi.1006019.s003.pdf]
