# Supplementary material for: iDREM: Interactive visualization of dynamic regulatory networks
Source: PLoS Comput Biol. 2018 Mar 14;14(3):e1006019. doi: 10.1371/journal.pcbi.1006019 (PMC5868853; doi:10.1371/journal.pcbi.1006019)
Supplement: S3 Fig — (A) The single-cell RNA-seq data. (B) Cluster the cells into different sub-types based on the expression profile. (C) Identify the signature genes (marker genes) for each cell type. (D) Intersect the marker genes (of specific cell-type) with the predicted paths/nodes in iDREM model to identify enriched paths/nodes. (E) This enables users to determine the cell type composition of the different nodes and paths and to infer whether specific changes observed are related to activation of TFs in existing cells or the formation of new cell types. (PDF) [file pcbi.1006019.s004.pdf]

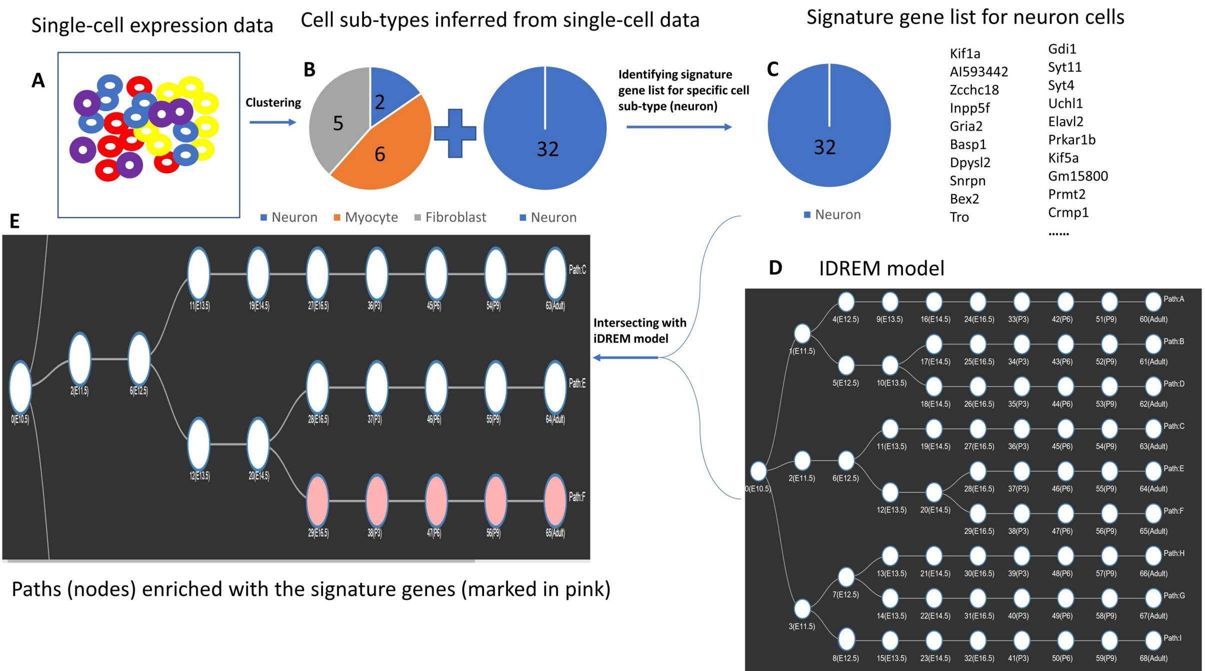
Single-cell expression data
Cell sub-types inferred from single-cell data
Signature gene list for neuron cells
A
Clustering
B
2
5
6
32
Neuron
Myocyte
Fibroblast
Neuron
Identifying signature gene list for specific cell sub-type (neuron)
C
32
Neuron
Kif1a
AI593442
Zcchc18
Inpp5f
Gria2
Basp1
Dpysl2
Snrpn
Bex2
Tro
Gdi1
Syt11
Syt4
Uchl1
Elavl2
Prkar1b
Kif5a
Gm15800
Prmt2
Crmp1
......
D
IDREM model
E
Intersecting with iDREM model
Path:A
Path:B
Path:C
Path:D
Path:E
Path:F
Path:G
Path:H
Path:I
0(E10.5)
Paths (nodes) enriched with the signature genes (marked in pink)
