# Supplementary material for: iDREM: Interactive visualization of dynamic regulatory networks
Source: PLoS Comput Biol. 2018 Mar 14;14(3):e1006019. doi: 10.1371/journal.pcbi.1006019 (PMC5868853; doi:10.1371/journal.pcbi.1006019)
Supplement: S4 Fig — I: only use miRNA and mRNA expression data; II: data used by I + time series proteomics data; III: the data used by I + the time series methylation data; IV: using all data presented in the study. (PDF) [file pcbi.1006019.s005.pdf]

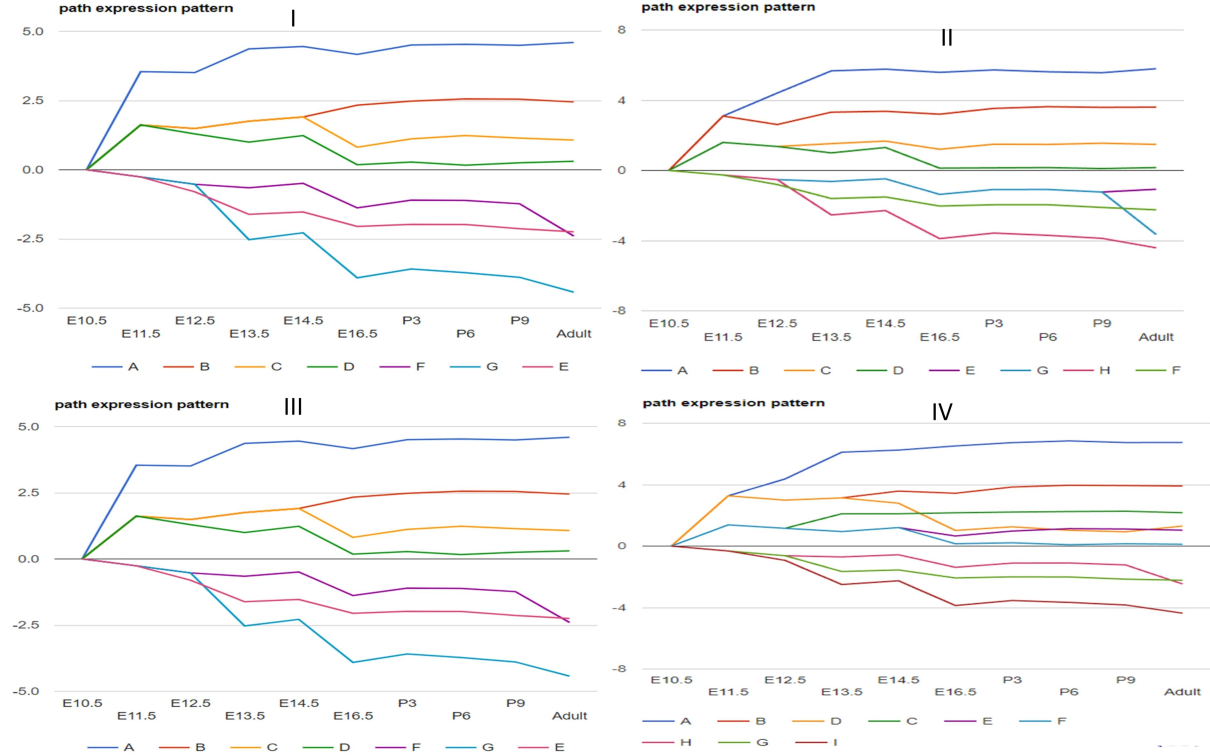
path expression pattern
I
5.0
2.5
0.0
-2.5
-5.0
E10.5
E11.5
E12.5
E13.5
E14.5
E16.5
P3
P6
P9
Adult
A
B
C
D
F
G
E
path expression pattern
II
8
4
0
-4
-8
E10.5
E11.5
E12.5
E13.5
E14.5
E16.5
P3
P6
P9
Adult
A
B
C
D
E
G
H
F
path expression pattern
III
5.0
2.5
0.0
-2.5
-5.0
E10.5
E11.5
E12.5
E13.5
E14.5
E16.5
P3
P6
P9
Adult
A
B
C
D
F
G
E
path expression pattern
IV
8
4
0
-4
-8
E10.5
E11.5
E12.5
E13.5
E14.5
E16.5
P3
P6
P9
Adult
A
B
D
C
E
F
H
G
I
