# Supplementary figures and images for: iDREM: Interactive visualization of dynamic regulatory networks
Source: PLoS Comput Biol. 2018 Mar 14;14(3):e1006019. doi: 10.1371/journal.pcbi.1006019 (PMC5868853; doi:10.1371/journal.pcbi.1006019)

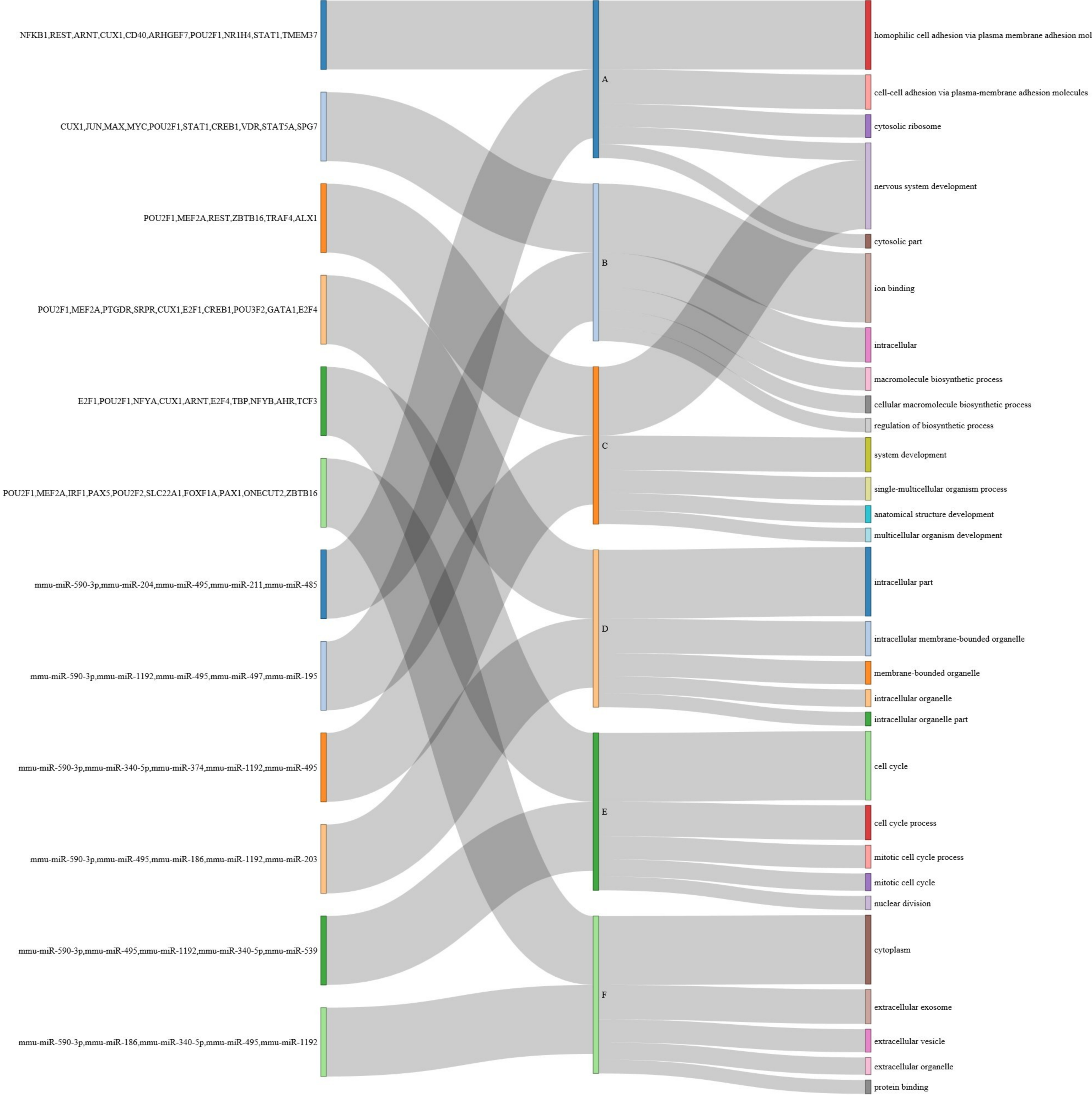

Supplement: S5 Fig — The Sankey Diagram shows the GO functions and regulators associated with each of the predicted paths. (PDF) [file pcbi.1006019.s006.pdf]

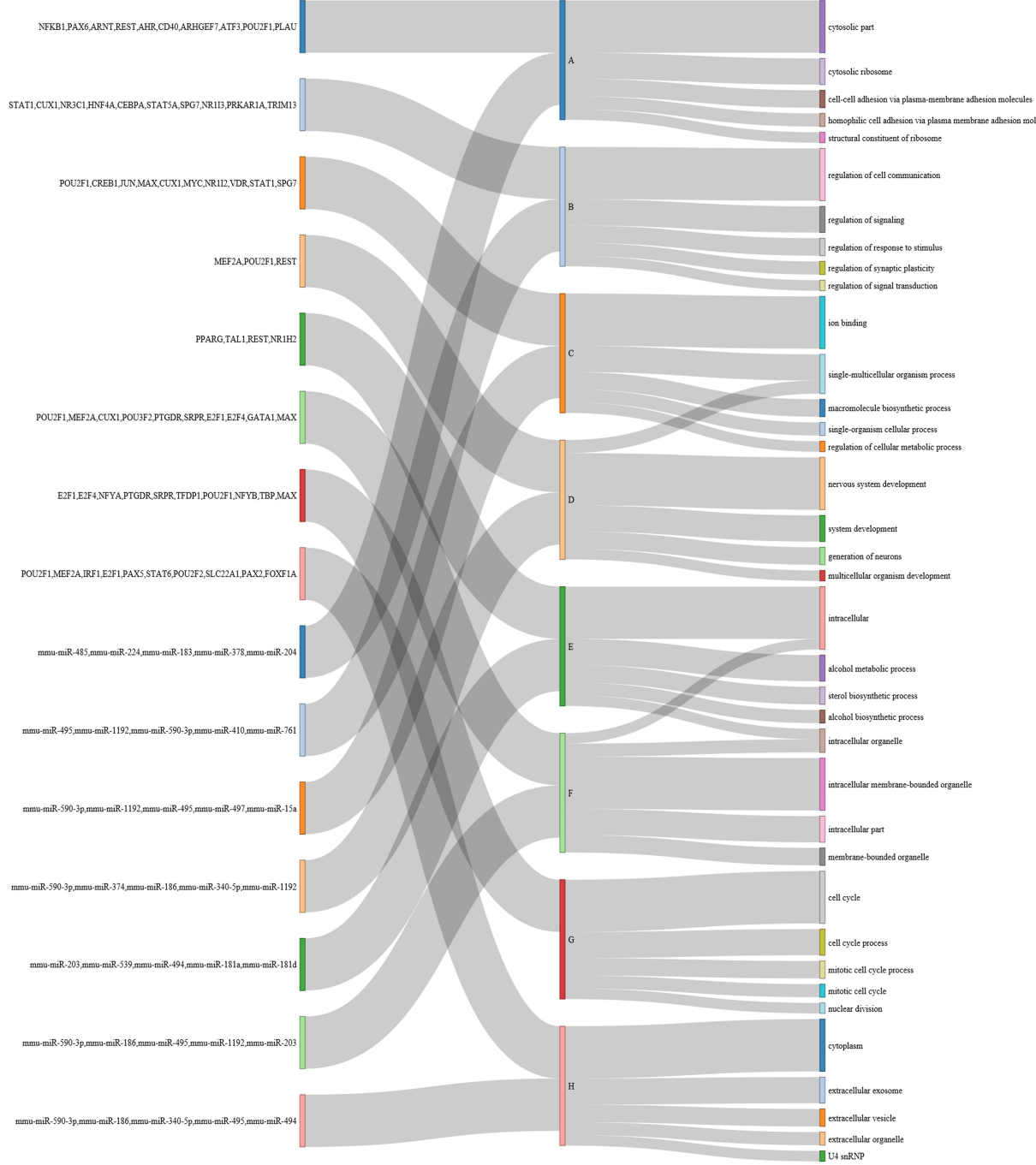

Supplement: S6 Fig — The Sankey Diagram shows the GO functions and regulators associated with each of the predicted paths. (PDF) [file pcbi.1006019.s007.pdf]

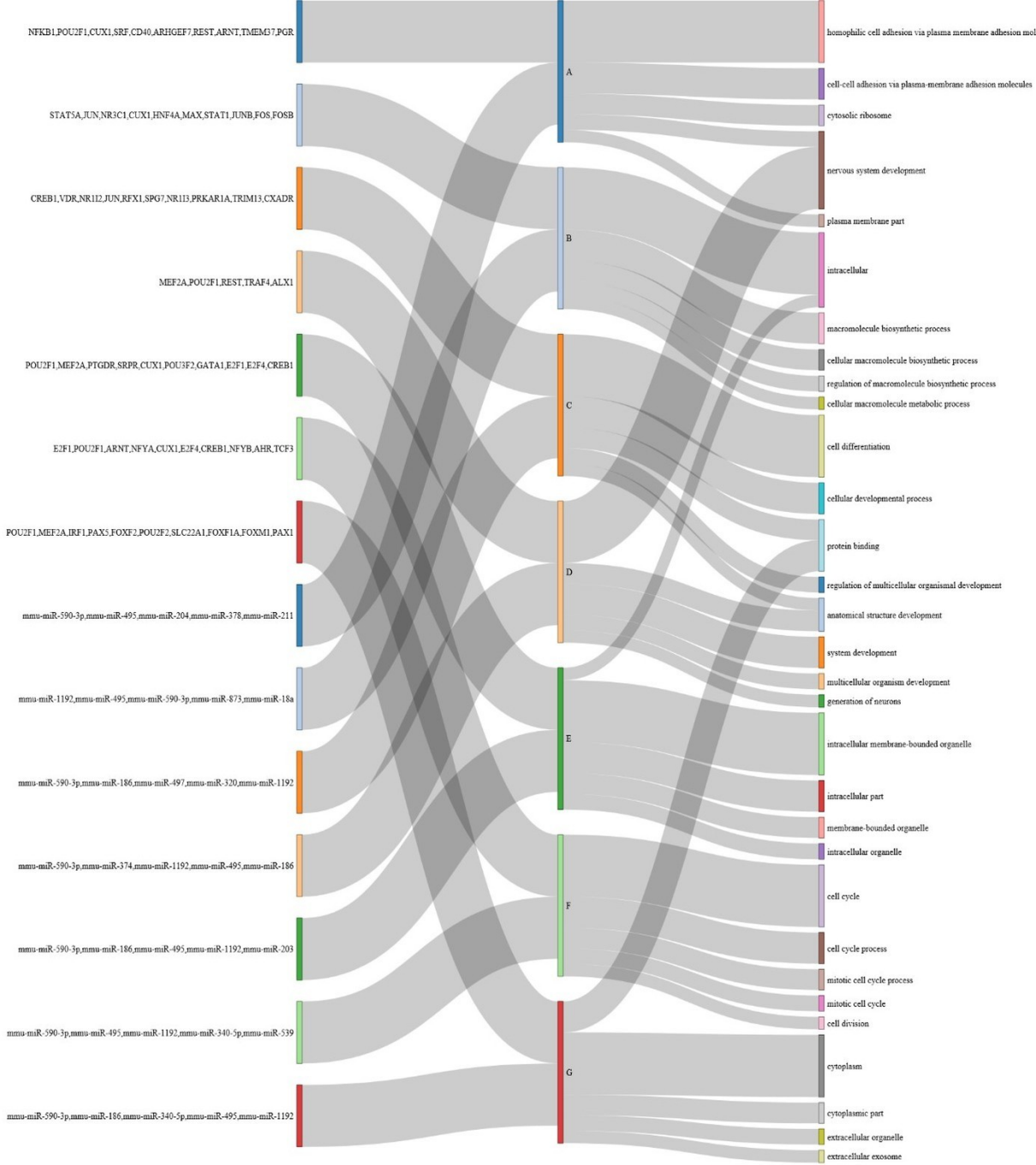

Supplement: S7 Fig — The Sankey Diagram shows the GO functions and regulators associated with each of the predicted paths. (PDF) [file pcbi.1006019.s008.pdf]

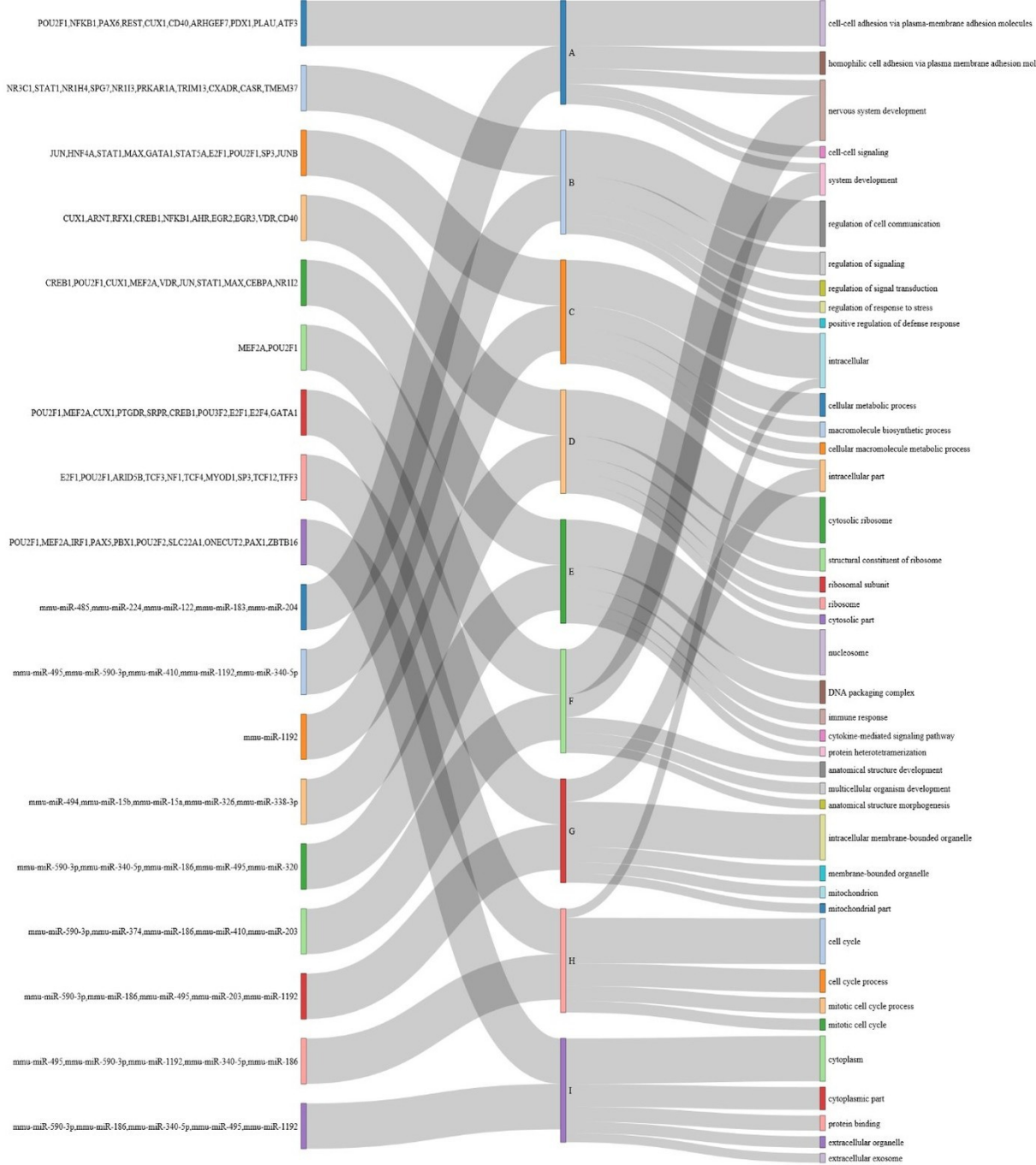

Supplement: S8 Fig — The Sankey Diagram shows the GO functions and regulators associated with each of the predicted paths. (PDF) [file pcbi.1006019.s009.pdf]
