# Supplementary material for: iDREM: Interactive visualization of dynamic regulatory networks
Source: PLoS Comput Biol. 2018 Mar 14;14(3):e1006019. doi: 10.1371/journal.pcbi.1006019 (PMC5868853; doi:10.1371/journal.pcbi.1006019)
Supplement: S1 Table — (PDF) [file pcbi.1006019.s010.pdf]

Table S 1: Mouse microglia development time points used in this paper

data	reference ID	Data type	time points									
mRNA expression data	PMID27338705	Microglia	E10.5	E11.5	E12.5	E13.5	E14.5	E16.5	P3	P6	P9	Adult
histone methylation data	PMID27338705	Microglia	-	-	E12.5	-	E14.5	E16.5	P3	-	-	Adult
miRNA expression	PMID15345052	whole brain	-	-	E12.5	-	-	E16.5(E17.5)	P3(P4)	-	-	Adult
proteomics data	PMID18283662	whole brain	E10.5 (E9.5)	E11.5	-	E13.5	-	-	-	-	-	-
