# Supplementary material for: iDREM: Interactive visualization of dynamic regulatory networks
Source: PLoS Comput Biol. 2018 Mar 14;14(3):e1006019. doi: 10.1371/journal.pcbi.1006019 (PMC5868853; doi:10.1371/journal.pcbi.1006019)
Supplement: S2 Table — (PDF) [file pcbi.1006019.s011.pdf]

Table S 2: Top GO terms associated with each path

Path	Top 3 GO terms
A	cell-cell adhesion via plasma-membrane adhesion molecules (p-value:2.96e-12; Bonferroni corrected p-value:3.46e-08)<br>homophilic cell adhesion via plasma membrane adhesion molecules(p-value:3.09e-12;Bonferroni corrected p-value:3.62e-08)<br>nervous system development (p-value:4.70e-10; Bonferroni corrected p-value:5.51e-6)
B	regulation of cell communication(p-value:2.59e-5; Bonferroni corrected p-value:0.304)<br>regulation of signaling(p-value:2.70e-5; Bonferroni corrected p-value:0.317)<br>regulation of response to stimulus(p-value:4.67e-5; Bonferroni corrected p-value:0.547)
C	intracellular (p-value:8.27e-15; Bonferroni corrected p-value:9.68e-11)<br>cellular metabolic process (p-value:4.62e-11; Bonferroni corrected p-value:5.41e-7)<br>macromolecule biosynthetic process (p-value:4.89e-11; Bonferroni corrected p-value:5.73e-7)
D	cytosolic ribosome (p-value:3.92e-34; Bonferroni corrected p-value:4.59e-30)<br>structural constituent of ribosome (p-value:6.66e-30; Bonferroni corrected p-value:7.80e-26)<br>ribosomal subunit (p-value:7.15e-30; Bonferroni corrected p-value:8.37e-26)
E	nuclear nucleosome (p-value:3.99e-7; Bonferroni corrected p-value:4.67e-3)<br>DNA packaging complex(p-value:1.56e-6; Bonferroni corrected p-value:0.0183)<br>immune response (p-value:2.15e-6; Bonferroni corrected p-value:0.0252)
F	nervous system development (p-value:8.61e-33; Bonferroni corrected p-value:1.01e-28)<br>system development (p-value:9.89e-32; Bonferroni corrected p-value:1.16e-27)<br>anatomical structure development (p-value:1.31e-28; Bonferroni corrected p-value:1.54e-24)
G	intracellular membrane-bounded organelle (p-value:1.17e-44; Bonferroni corrected p-value:1.37e-40)<br>intracellular part(p-value:5.58e-44; Bonferroni corrected p-value:6.54e-40)<br>membrane-bounded organelle (p-value:3.10e-43; Bonferroni corrected p-value:3.64e-39)
H	Cytoplasm (p-value:5.54e-9; Bonferroni corrected p-value:6.50e-5)<br>cytoplasmic part (p-value:2.20e-8; Bonferroni corrected p-value:2.58e-4)<br>protein binding (p-value:5.67e-8; Bonferroni corrected p-value:6.64e-4)
