# Supplementary material for: iDREM: Interactive visualization of dynamic regulatory networks
Source: PLoS Comput Biol. 2018 Mar 14;14(3):e1006019. doi: 10.1371/journal.pcbi.1006019 (PMC5868853; doi:10.1371/journal.pcbi.1006019)
Supplement: S3 Table — (PDF) [file pcbi.1006019.s012.pdf]

Table S 3: Supported regulating factors predicted by iDREM

TF	p-value	reported regulating stages	predicted regulating stages by iDREM
Rxrb	-	E12.5-Adult	-
Fli1	2.95e-47	E14-Adult	E12.5- Adult
Egr1	4.23e-58	E14-Adult	E14.5-Adult
Fos	1.24e-58	E14-Adult	E12.5-Adult
Mafb	-	Adult	-
Mef2a	2.06e-48	E16.5, P3 , Adult	E13.5-Adult
Jun	1.74E-67	E12.5, E14, Adult	E12.5-Adult
