# Supplementary material for: iDREM: Interactive visualization of dynamic regulatory networks
Source: PLoS Comput Biol. 2018 Mar 14;14(3):e1006019. doi: 10.1371/journal.pcbi.1006019 (PMC5868853; doi:10.1371/journal.pcbi.1006019)
Supplement: S4 Table — (PDF) [file pcbi.1006019.s013.pdf]

Table S 4: Regulator comparison for models using different sets of input data

Regulator	model I	model II	model III	model IV
Rxrb	No	No	No	No
Fli1	No	No	Yes	Yes
Egr1	No	No	No	Yes
Fos	Yes	Yes	Yes	Yes
Mafb	No	No	No	No
Mef2a	Yes	Yes	Yes	Yes
Jun	Yes	Yes	Yes	Yes
Cd40	Yes	Yes	Yes	Yes
Smad1	Yes	Yes	Yes	Yes
Traf4	No	No	No	Yes
number of predicted verified TFs	5	5	6	8
